# Experimental analysis of diverse actin-like proteins from various magnetotactic bacteria by functional expression in *Magnetospirillum gryphiswaldense*

**DOI:** 10.1128/mbio.01649-23

**Published:** 2023-10-12

**Authors:** Ram Prasad Awal, Frank D. Müller, Daniel Pfeiffer, Caroline L. Monteil, Guy Perrière, Christopher T. Lefèvre, Dirk Schüler

**Affiliations:** 1 Department of Microbiology, Universitat Bayreuth, Bayreuth, Germany; 2 Aix-Marseille Université, CEA, CNRS, Institute of Biosciences and Biotechnologies of Aix-Marseille, Saint-Paul-lez-Durance, France; 3 Laboratoire de Biométrie et Biologie Evolutive, Université Claude Bernard-Lyon 1, Villeurbanne, France; University of California, Berkeley, California, USA

**Keywords:** actin-like, MamK, Mad28, cytoskeleton, magnetoskeleton, magnetotactic bacteria

## Abstract

Magnetotactic bacteria (MTB) produce magnetosomes, which are sensory organelles consisting of nanocrystals of a magnetic iron mineral enclosed by membranes. In the well-characterized *Magnetospirillum* species of the *Alphaproteobacteria*, magnetosomes align and form highly ordered chains along filaments that consist of the bacterial actin homolog MamK. The MamK protein is part of a multi-component “magnetoskeleton” that controls the concatenation, positioning, and partitioning of the magnetosome chains (MCs) which serve as cellular compass for efficient navigation in the Earth’s magnetic field. MamK is highly conserved in all MTB; however, it is unknown whether its magnetoskeletal function is preserved, especially in those MTB which exhibit distinct and more complex architectures of MCs and often contain additional putative magnetoskeletal constituents such as the actin-like protein Mad28 with as yet-unknown functions. Here, we studied the ability of magnetosome-associated actins from a wide range of diverse MTB to rescue well-characterized magnetoskeleton mutants of the model *Magnetospirillum gryphiswaldense*. We found that MamK orthologs from *Alpha-*, *Delta-*, *Candidatus Etaproteo-*, and *Nitrospirota*-MTB as well as a resurrected MamK LUCA version restored MC assembly to varying degrees and exhibited filamentous localization in *M. gryphiswaldense* and *E. coli*. We also identified a novel magnetosome-related protein from the magnetotactic alphaproteobacterium *Magnetovibrio blakemorei* that substitutes the function of the well-characterized MamJ protein as a molecular adaptor tethering magnetosomes to MamK filaments. Moreover, we demonstrate that Mad28 orthologs from *Thermodesulfobacteriota* and *Nitrospirota* are actin-like proteins that can functionally complement *mamK* mutants of *M. gryphiswaldense* and which form filamentous structures *in vivo and in vitro*.

**IMPORTANCE:**

To efficiently navigate within the geomagnetic field, magnetotactic bacteria (MTB) align their magnetosome organelles into chains, which are organized by the actin-like MamK protein. Although MamK is the most highly conserved magnetosome protein common to all MTB, its analysis has been confined to a small subgroup owing to the inaccessibility of most MTB. Our study takes advantage of a genetically tractable host where expression of diverse MamK orthologs together with a resurrected MamK LUCA and uncharacterized actin-like Mad28 proteins from deep-branching MTB resulted in gradual restoration of magnetosome chains in various mutants. Our results further indicate the existence of species-specific MamK interactors and shed light on the evolutionary relationships of one of the key proteins associated with bacterial magnetotaxis.

## INTRODUCTION

Actin-like proteins are among the cytoskeletal core components in all domains of life, and because of their propensity to form dynamic filaments, they fulfill numerous and often essential functions as structural and cytomotive elements (1 – 3). Bacterial actin-like proteins clade apart from eukaryotic actins (4) but share a similar tertiary structure and a conserved nucleotide-binding pocket with their eukaryotic counterparts, and they are functionally distinct (5 – 10). For example, the bacterial actin proteins MreB, FtsA, and ParM have roles in cell shape determination by controlling cell wall synthesis (11), cytokinesis (12), or plasmid DNA segregation (13, 14), respectively. Another well-characterized example is the MamK protein present in all described magnetotactic bacteria (MTB). Phylogenetically, MamK proteins form a clade distinct from other bacterial actins (7, 15). MamK was first discovered in species of the genus *Magnetospirillum* (*Alphaproteobacteria*) (15 – 19) where it helps to assemble and organize their intracellular nanocrystals of magnetite (Fe_3_O_4_) into linear magnetosome chains (MCs) that align the cells in the geomagnetic field. MamK from both *Magnetospirillum magneticum* AMB-1 and *Magnetospirillum gryphiswaldense* (*Mgryph*) MSR-1 was shown to form filaments *in vitro* (5, 20, 21), which is dependent on ATP binding and hydrolysis. *In vivo*, MamK from AMB-1 and *Mgryph* localize in cell-spanning filamentous patterns as seen when fused to fluorescent proteins, whereby linear signals in AMB-1 (15, 22) and linear-to-helical signals in *Mgryph* (23) were identified. Cryo-electron tomography revealed that the MamK filaments within cells form a spindle-like structure, which extends from one pole of the cell to the other (15, 24). However, MamK filaments do not just play a role as static scaffold. Analyses of active site mutants led to the perception that nucleotide hydrolysis and subunit turnover result in filament dynamics, which is conveyed to magnetosomes and results in dynamically positioned MCs. For example, in *Mgryph*, MamK acts in the formation of one or two contiguous MCs located precisely at mid-cell, which results in equal partitioning of magnetosomes upon cytokinesis, and pole-to-mid-cell treadmilling of MamK filaments was shown to be required for rapid repositioning of split MCs from the cell pole to mid-cell thereafter (25). Deletion of *mamK* did not entirely abolish MC formation but resulted in off-centered shorter, fragmented, and ectopic MCs (23, 26). This chain fragmentation as a hallmark of the ∆*mamK_Mgryph_* mutant is thought to occur because the dynamic cytomotive action of magnetosome-attached MamK polymers is missing (27). In contrast to *Mgryph*, AMB-1 WT cells form MCs which appear fragmented due to the presence of empty vesicles among mature crystal-containing vesicles (15, 28, 29). Deletion of *mamK* in AMB-1 resulted in a disorganized MC and the loss of magnetosome-associated filaments (15). In both organisms, the function of MamK depends on specific interactors, such as the acidic protein MamJ (16, 18, 30). MamJ is essential for MC formation in *Mgryph* by acting as a connector that attaches magnetosomes to MamK filaments, and its deletion resulted in chain collapse and clustered magnetosomes (24, 31). In AMB-1, MamJ and its homolog LimJ are required for the turnover of MamK filament subunits; however, their deletion resulted in a less drastic phenotype than in *Mgryph* (30). Furthermore, a recent study by Toro-Nahuelpan et al. (27) reported that magnetosomes in *Mgryph* are linked via MamJ to scaffolds formed not only by MamK but also by the magnetosome-associated protein MamY, which together constitute the tripartite “magnetoskeleton.” MamY localizes along the geodetic line within the spiral cell body and helps MC to maintain a straight configuration along the inner positive cell curvature of helical cells, thus aligning the magnetic dipole parallel to the cell‘s axis of motility (27, 32). In the Δ*mamY_Mgryph_* mutant, the MamK-bound MC is intact but mislocalized to the negative cell curvature, and the effectivity of magnetotaxis is impaired (33). The combined deletion of *mamK* and *mamY* in *Mgryph* resulted in a complete loss of MCs reminiscent of ∆*mamJ_Mgryph_* (27, 32).

Genomic and metagenomic studies revealed that MamK is part of the only few signature genes (*mamABIKMQ*) present in the genome of all cultured and uncultured MTB known so far (34 – 36). Among all known magnetosome-related proteins, MamK orthologs display sequence similarities that range from 60%–70% between closely related species to 20%–30% when MamK*_Mgryph_* is compared to distantly related MTB from *Nitrospirota* [former *Nitrospirae* (37)] and other groups (34, 36, 38), indicating that *mamK* is the single most conserved magnetosome gene across MTB (15, 31, 34, 35, 39 – 42). Despite this high conservation, it is not yet clear whether the cytoskeletal function of MamK is preserved between various MTB or has diverged over time. For example, some MTB such as AMB-1, *Magnetovibrio blakemorei* MV-1, and the gammaproteobacteria strain SS-5 and BW-2 have been found to contain two divergent copies of *mamK* in their magnetosome biosynthesis gene clusters (20, 38). In addition, MamJ, which is one of the main MamK interactors in magnetospirilla, seems to be missing outside this group suggesting that MamK dynamics and MC assembly might be controlled differently. A somewhat distinct or more complex mechanism of MC assembly is also suggested by the observation that the number and subcellular arrangement of MCs increasingly diverges in MTB with phylogenetic distance to magnetospirilla. For example, double or multiple MCs are observed in many magnetococci (43, 44) and in MTB from the *Nitrospirota* (40). However, the function of MamK orthologs from these and most other MTB has not yet been experimentally tested due to their inaccessibility. Moreover, MTB belonging to *Thermodesulfobacteriota*, *Nitrospirota*, and *Candidatus Omnitrophota* contain a further putative actin-like protein in addition to MamK, which was termed Mad28 (magnetosome associated deep branched). It has been reported that Mad28 in the *Nitrospirota* Mbav contains a conserved actin-like domain and that Mad28 from the *Thermodesulfobacteriota* and *Nitrospirota* form a clade distinct from other bacterial actin-like proteins (4, 38). The *mad28* gene is located in close proximity to magnetosome-related genes (34, 38) and can be present in multiple divergent copies, which again may be associated with multiplicity of MCs in these MTB. The deltaproteobacterium *Desulfamplus magnetovallimortis* BW-1, on the other hand, contains only one copy of *mad28* besides *mamK* and forms only a single magnetosome chain of magnetite or greigite crystals (38). Despite its ubiquitous occurrence in *Thermodesulfobacteriota*- and *Nitrospirota*-MTB, Mad28 has not been experimentally analyzed so far, again due to a lack of genetically accessible or cultured models from those groups. As a result, its function, polymeric structures, and any potential interaction with MamK have remained elusive.

A powerful method for elucidating the function of foreign proteins from inaccessible donors is to express them in a surrogate host. This approach was recently demonstrated for several conserved magnetosome genes from selected MTB donors, which rescued their orthologous mutants in the tractable model *Mgryph* (45). Here, we took advantage of the model *Mgryph* as a heterologous host and its well-characterized ∆*mamK* and ∆*mamKY* mutants, where we expressed *mamK* and *mad28* orthologs covering a wide phylogenetic range of MTB and donor strains that exhibit various shapes and arrangements of crystals and comprising the following representatives: the closely related strain AMB-1 unlike *Mgryph* forms fragmented MCs (15). The vibriod marine MV-1 from the *Alphaproteobacteria* produces a single linear MC of about 10 elongated magnetite crystals (46, 47). The marine *Magnetococcus marinus* MC-1 produces a single chain of 10–14 elongated magnetite crystals (47). *Solidesulfovibrio magneticus* RS-1 is a freshwater sulfate-reducing deltaproteobacterium that assembles 12–15 irregular bullet-shaped magnetite crystals in a poorly ordered chain (48, 49). Another sulfate-reducing deltaproteobacterium *Desulfamplus magnetovallimortis* BW-1, however, produces magnetite and/or greigite nanocrystals, aligned in one or more MCs (50). The uncultured *Candidatus* Magnetobacterium bavaricum *Mbav* and *Ca*. Magnetobacterium casensis MYR-1 from the *Nitrospirota* synthesize between 600 and 1,000 bullet-shaped magnetite crystals that are arranged in several parallel chains, which in turn form rope-like strands or bundles (41, 51). Metagenomic *mamK* from the uncultured *Magnetococcus* bacterium DCbin4 belonging to *Candidatus* Etaproteobacteria and HCHbin1 from *Nitrospirota*, both with unknown morphologies and MC configurations, were also included (34).

We analyzed the localization patterns of the heterologous proteins from different MTB phyla and their potential to complement the *mamK* and *mamKY* mutants in *Mgryph*. We show that MamK orthologs from foreign MTB can complement deletion mutants to different degrees and display similar localization patterns, suggesting that they are functionally equivalent. These analyses were complemented by functional expression of a putative ancestral MamK version, which was resurrected from many extant MamK (Fig. S1; Table S1A). Furthermore, we identified Mg-1g50 as a novel magnetosome-related protein in MV-1 that seems to substitute for MamJ’s function in the assembly of MCs. In addition, we provide experimental evidence that the Mad28 protein is a novel magnetosome-related bacterial actin-like protein in MTB.

## RESULTS

### Foreign MamK orthologs restore magnetosome chain formation in Δ*mamK*_Mgryph_ and Δ*mamKY*_Mgryph_ magnetoskeleton mutants to different degrees

**Fig 1:**
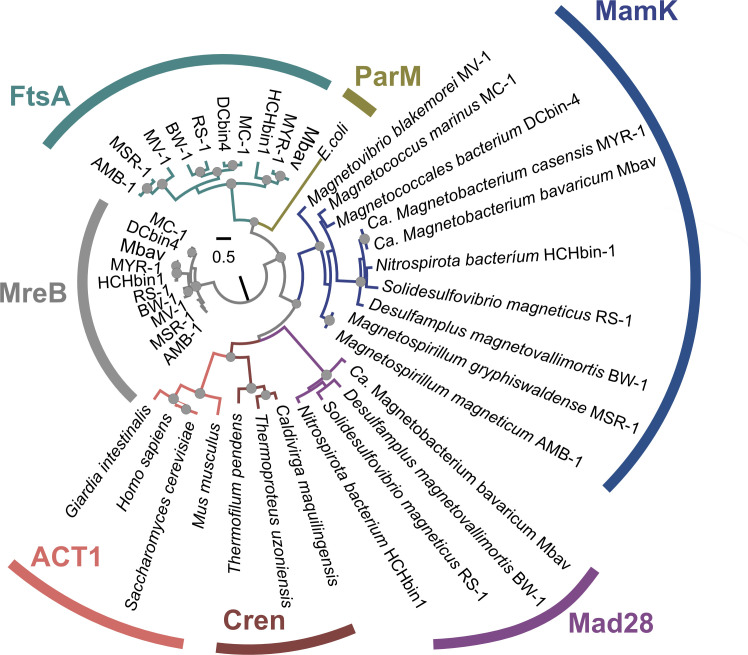
Maximum-likelihood phylogenetic tree of the actin ATPase protein family showing that Mad28 proteins of magnetotactic *Thermodesulfobacteriota* and *Nitrospirota* form a distinct clade compared to other actin-related proteins like MamK, MreB, and FtsA found in magnetotactic bacteria. The tree was drawn to scale, and circles represent statistical support estimated from 500 non-parametric bootstraps. Only significant values above 80 are shown. The sequence database was inspired from that of Monteil and colleagues (4) where accession numbers of crenactin family (Cren) and eukaryotic actin 1 (ACT1) can be found. Here, the MreB family was used to root the tree, since it forms the most ancestral monophyletic group of Actin like proteins based on Ettema and colleagues (10).

First, we studied whether the selected MamK orthologs, which share between 71% and 98% sequence similarity and 35% and 95% identity to MamK*_Mgryph_* (Table S1B; Fig. 1), can substitute the native MamK function in *Mgryph*. To this end, we placed these genes under control of the promoter P*_mamDC_*_45_ from *Mgryph* and transferred them into the magnetoskeleton mutants Δ*mamK_Mgryph_* and Δ*mamKY_Mgryph_* by Tn5-transposon-mediated random genome insertion. We then analyzed the degree of complementation by scoring the magnetosome organization and positioning based on transmission electron microscopy (TEM) imaging, as well as the restoration of the cellular magnetic orientation by *C*_mag_ determination.

**Fig 2:**
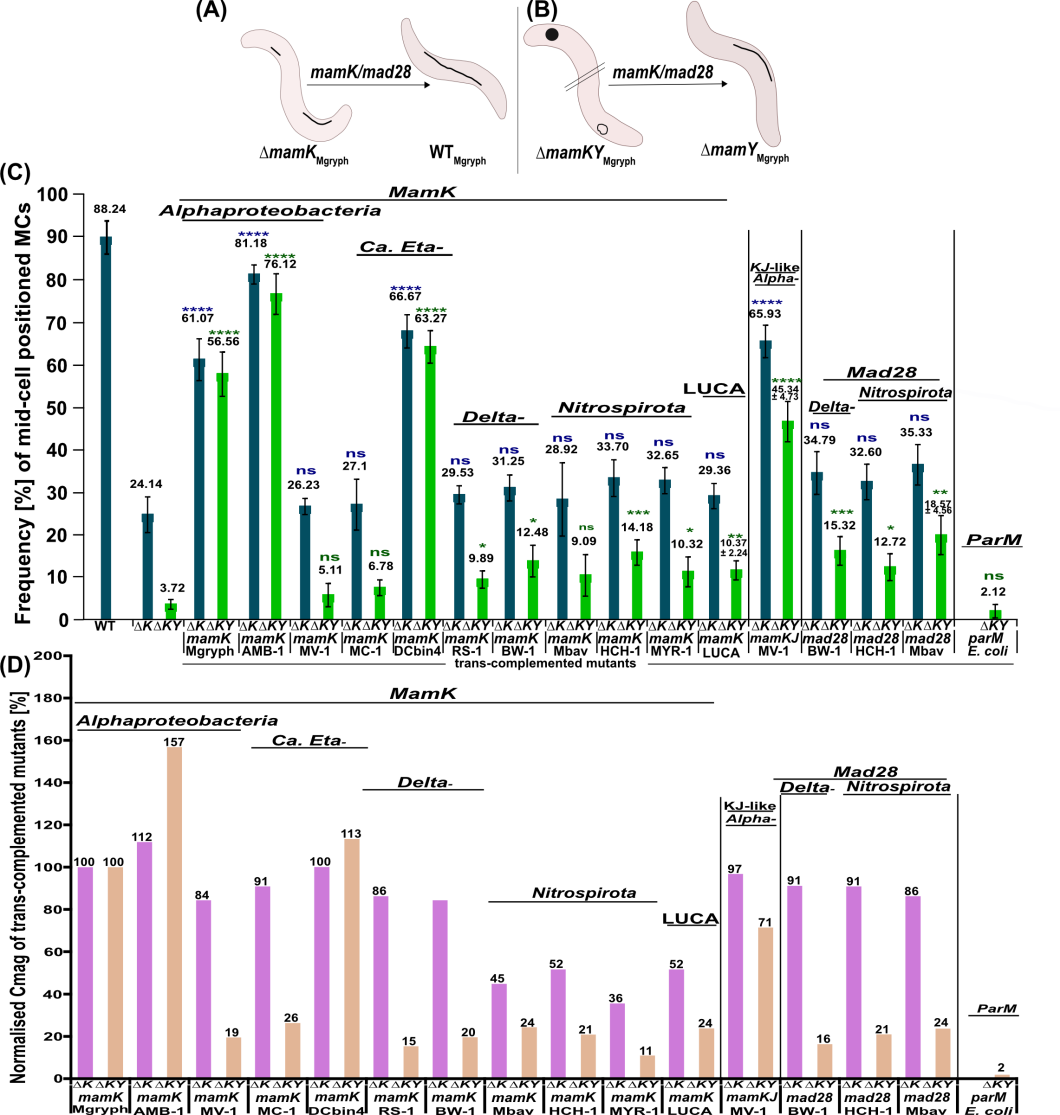
(**A and B**) Schematic representation of magnetosome organization in WT*_Mgryph_* and the magnetoskeleton mutants ∆*mamK_Mgryph_*, ∆*mamKY_Mgryph_*, and ∆*mamY_Mgryph_*. WT, magnetosomes organized in straight chains that run along the geodetic cell axis; ∆*mamK_Mgryph_*, short, fragmented, off-center chains; ∆*mamKY_Mgryph_*, agglomerated magnetosomes or magnetic flux-closed rings; ∆*mamY_Mgryph_*, magnetosome chain at the negative inner cell curvature. Arrows indicate expected reconstitution of magnetosome chain organization in case of successful trans-complementation with *mamK* from diverse MTB in ∆*mamK_Mgryph_* (**A**) and in ∆*mamKY_Mgryph_* (**B**). (**C**) Degree of complementation as inferred from MC organization in ∆*mamK_Mgryph_* and ∆*mamKY_Mgryph_*. Blue bars, frequency of magnetosome chains positioned at mid-cell in WT*_Mgryph_*, ∆*mamK_Mgryph_*, and ∆*mamK_Mgryph_* that was complemented with the indicated genes. Green bars, frequency of magnetosome chains (>10 particles, resembling ∆*mamY_Mgryph_* phenotype) in ∆*mamKY_Mgryph_* and ∆*mamKY_Mgryph_* that was trans-complemented with the indicated genes. The numbers of analyzed cells (N) correspond to >200. All values are expressed as mean ± standard error of the mean (SEM). Statistical significance is indicated as follows: *P ≤ 0.05, **P ≤ 0.01, ***P ≤ 0.001, ****P ≤ 0.0001, and non-significant (ns) when *P* > 0.05. Statistical significance was assessed using a non-paired *t*-test between the control strains (Δ*mamK_Mgryph_* in blue and Δ*mamKY_Mgryph_* in green) and the corresponding trans-complemented mutants in the control strains. (**D**) Relative magnetic response (*C_mag_*) of complemented ∆*mamK_Mgryph_* and ∆*mamKY_Mgryph_* strains. The *C_mag_* of *Mgryph* strains that were complemented with the native allele was set at 100%, and the *C*_mag_ of the strains with diverse *mamKs*, *mamK*_LUCA_, *mamKJ*-like_MV-1_, *mad28*, and *parM* genes was normalized to the respective value to obtain the relative *C*_mag_ as indicator for functional complementation. The values of *C_mag_* were derived from four independent Tn5 insertion mutants.

To establish the base for our measurements properly, we first re-assessed the Δ*mamK_Mgryph_* and Δ*mamKY_Mgryph_* deletion mutants. As previously reported by (23), Δ*mamK_Mgryph_* exhibited short, off-center, fragmented, and ectopic MCs and a reduced *C*_mag_ of 0.93 (WT*_C_*_mag_, 1.43) (Fig. 2A; Fig. S3 and S4C). For Δ*mamKY_Mgryph_*, we observed cells with a complete loss of the MC, but magnetosomes were aggregated or localized in magnetic flux-closed rings as reported before (27) (Fig. 2B). However, we also found that few of the Δ*mamKY_Mgryph_* cells contained short (<5 particles) and long MC (>10 particles) shifted toward the negative cell curvature, rather resembling the Δ*mamY_Mgryph_* single deletion phenotype (Fig. S4D). To address methodological factors that are inherent to our experimental approach, we next re-inserted the native *mamK_Mgryph_* gene and determined the degree of complementation. Transfer of the native *mamK_Mgryph_* into Δ*mamK_Mgryph_* increased the *C*_mag_ from 0.93 to 1.37 (Fig. S3), and cells predominantly showed mid-cell linear contiguous MCs (~61%) (Δ*mamK_Mgryph_*, ~24%) (Fig. 2C; Fig. S4AC), indicating overall functional complementation. Less frequently, we still detected some ectopic (at one or both cell poles) or up to four fragmented MCs (Fig. S4C) resembling the Δ*mamK_Mgryph_* mutant phenotype, which might be due to non-native MamK expression levels. Likewise, transfer of *mamK_Mgryph_* into Δ*mamKY_Mgryph_* restored the Δ*mamY_Mgryph_* phenotype, i.e., MCs were again assembled, but shifted to the negative cell curvature, and the *C*_mag_ increased to 1.12 similar to that described in reference (27) (Fig. 2B; Fig. S3). The frequency of long MC with >10 particles increased from ~4% to 57% in (Fig. 2C; Fig. S4D), and the average *C*_mag_, to 0.74 (~290% of Δ*mamKY_Mgryph_*; *C_mag_* of Δ*mamKY_Mgryph_* = 0.19) (Fig. 2D; Fig. S3). Besides long MCs, a proportion of complemented Δ*mamKY_Mgryph_* cells also contained short chains and magnetosome rings but at lower frequency as in the parent Δ*mamKY_Mgryph_* (Fig. S3B and D).

The *C*_mag_ values of Δ*mamK_Mgryph_* and Δ*mamKY_Mgryph_* strains, each complemented with the native *mamK_Mgryph_*, were averaged and set as reference (100%) to calculate the relative degree of complementation by foreign *mamK*s, which we transformed next. Trans-complementation of Δ*mamK_Mgryph_* with *mamK* from AMB-1 and DCbin4 resulted in a *C*_mag_ higher or similar to Δ*mamK_Mgryph_*::*mamK_Mgryph_* (Fig. 2D; Fig. S3), and the frequency of non-fragmented mid-cell MC was increased to ~81% and ~67%, respectively, demonstrating a significant enhancement compared to that of Δ*mamK_Mgryph_* (Fig. 2C), indicating a substantial functional substitution of MamK*_Mgryph_*. Trans-complementation of Δ*mamKY_Mgryph_* with *mamK*_AMB1_ and *mamK*_DCbin4_ increased the frequency of long MCs (>10 particles) to ~76% and ~63% in the populations, respectively, showing a significant enhancement compared to that of Δ*mamKY_Mgryph_* (Fig. 2C). Upon expression of *mamK*_AMB1_ in Δ*mamKY_Mgryph_*, the *C*_mag_ values were increased by 57% of the *C*_mag_ relative to the Δ*mamKY_Mgryph_* strain trans-complemented with *mamK*_Mgryph_, whereas *mamK*_DCbin4_ expression in the same genetic background resulted in a 13% higher *C*_mag_ (Fig. 2D), again suggesting functional equivalence to MamK*_Mgryph_*.

MamK from MV-1, MC-1, RS-1, BW-1, Mbav, HCHbin-1, and MYR-1 also increased the frequency of mid-cell non-fragmented long MCs in Δ*mamK_Mgryph_* but to a considerably lower degree (~26%–34%) (Fig. 2C). *C*_mag_ of these trans-complemented strains reached only ~36%–86% of *mamK_Mgryph_* (Fig. 2D), with a comparable or even higher frequency of cells with ectopic MCs (Fig. S4C). Likewise, *mamK* genes in Δ*mamKY_Mgryph_* only slightly increased the frequency of long chains (>10 particles, ~5%–14%) compared to the uncomplemented Δ*mamKY_Mgryph_* (~4%) (Fig. 2C). These increases were statistically significant at varying degrees, except for mutants with *mamK* from MV-1, MC-1, and Mbav (Fig. 2C). The complemented mutants had a *C*_mag_ of ~11%–26% of *mamK_Mgryph_* (Fig. 2D).

To test whether actin-like proteins from non-MTB and not involved in magnetotaxis may have an effect on magnetosome localization, for example, by just restraining their subcellular position sterically, we included ParM from *E. coli* and transferred this gene into Δ*mamK_Mgryph_* and Δ*mamKY_Mgryph_*. Whereas transfer of *parM_E. coli_* into Δ*mamK_Mgryph_* failed for unknown reason, the transfer of *parM _E. coli_* into Δ*mamKY_Mgryph_* resulted in trans-complemented mutant strain, which, however, did not exhibit a noticeable increase in the occurrence of long mid-cell positioned MCs, and the magnetosome organization largely remained identical to that of the control strain Δ*mamKY_Mgryph_* (Fig. 2C; Fig. S4D). Moreover, the trans-complemented mutant displayed a *C*_mag_ value equivalent to only 2% of that observed in the Δ*mamKY_Mgryph_::mamK_Mgryph_* strain suggesting the lack of functional restoration (Fig. 2D; Fig. S3).

### MamK proteins localize in a linear pattern

**Fig 3:**
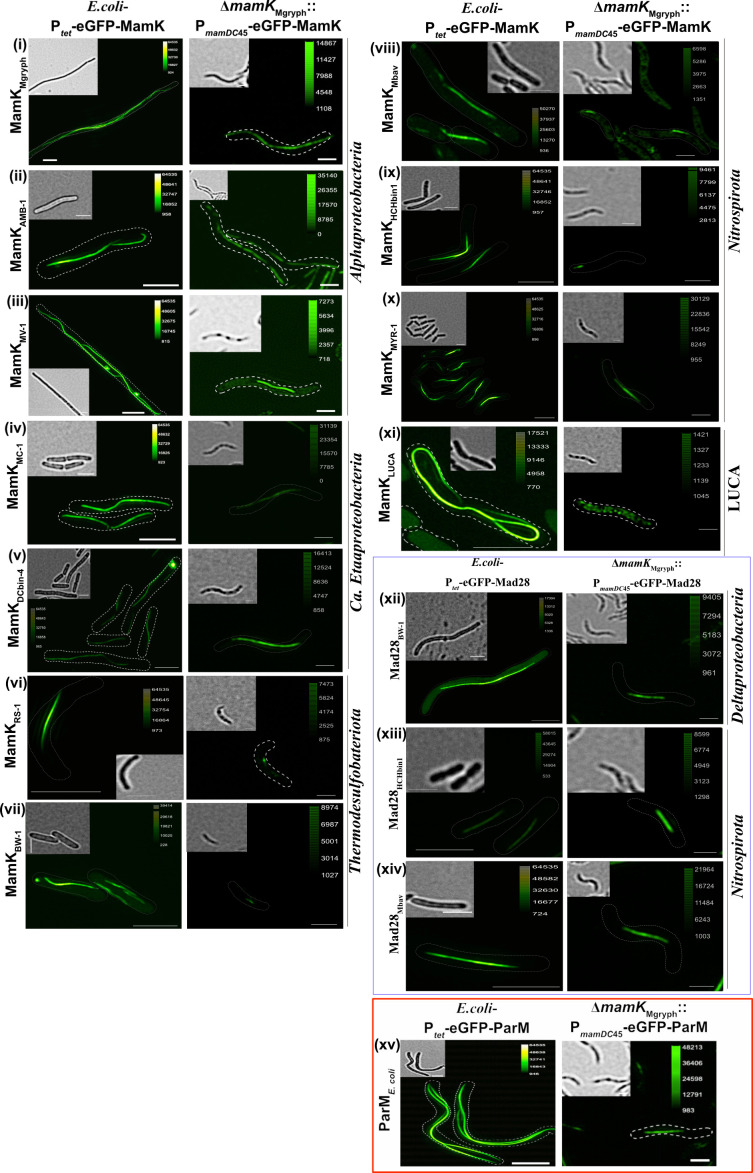
Three-dimensional structured illumination fluorescence microscopy micrographs (maximum intensity projection, brightfield as inset) of *E. coli* and *Mgryph* cells expressing EGFP-MamK/EGFP-Mad28 from diverse MTB and EGFP-ParM*_E.coli_*. Gene expression in *E. coli* was induced with anhydrotetracycline for 6 hours (50 ng/mL). In Δ*mamK_Mgryph_*, genes were constitutively expressed from P*_mamDC_*_45_. Scale bars correspond to 1 µm.

To analyze the intracellular localization of the various MamK orthologs, they were N-terminally fused to enhanced green fluorescent protein (EGFP) expressed from low copy number plasmids under control of the tetracycline inducible promoter P*_tet_* in *E. coli* and in *Mgryph* after chromosomal insertion via Tn5 transposition from the endogenous constitutive promoter P*_mamDC_*_45_. As reported previously, the control fusion proteins EGFP-MamK*_Mgryph_* and EGFP-MamK_AMB-1_ localized as a continuous line from pole to pole in both *E. coli* and Δ*mamK_Mgryph_* (20, 23) (Fig. 3I). EGFP-MamK_MV-1_ localized from pole to pole as linear structure in *E. coli*, whereas only a short filamentous signal at mid-cell was observed in Δ*mamK_Mgryph_* (Fig. 3III). Localization of EGFP-MamK from *Candidatus Etaproteobacteria* (MC-1 and DCbin4) in *E. coli* and Δ*mamK_Mgryph_* appeared as straight lines extending along the longitudinal cell axis (Fig. 3iv and v). EGFP-MamK from *Thermodesulfobacteriota* RS-1 and BW-1 and *Nitrospirota* Mbav, HCHbin-1, and MYR-1 localized as a long filament in *E. coli* as well (Fig. 3VI–X). These MamK fusions formed short linear strands or patches in Δ*mamK_Mgryph_* (Fig. 3VI–ix), except for EGFP-MamK_MYR-1_, which localized as short filament at mid-cell (Fig. 3X). The EGFP-ParM*_E. coli_* control localized as filamentous structure in both *E. coli* and Δ*mamK_Mgryph_* (Fig. 3xv).

### Complementation by MamK_MV-1_ depends on a novel MamJ-like protein

While MamK from AMB-1 and DCbin4 restored the *C*_mag_ similar to MamK*_Mgryph_*, MamK from MV-1 and the more distantly related strains RS-1, BW-1, Mbav, HCHbin1, and MYR-1 increased the *C*_mag_ only slightly. This might be due to reduced interaction efficiency of the divergent foreign proteins with endogenous magnetoskeletal or magnetosomal constituents in *Mgryph*. One of those is MamJ, which in magnetospirilla acts as a molecular adaptor that tethers magnetosomes to MamK filaments and is necessary for tuning of MamK turnover rates (27). Because of this essential function for MC formation, it is likely that less similar or unrelated proteins substitute MamJ in MTB outside magnetospirilla, where MamJ orthologs have not been identified. To test this hypothesis, we first searched for potential as-yet-undetected MamJ-like homologs in the genome of the magnetotactic alphaproteobacterium MV-1. As before, a BLASTP similarity search using the complete protein sequence of MamJ*_Mgryph_* as a query failed to generate any significant hit. However, full-length MamJ*_Mgryph_* contains the hypervariable non-essential central acidic repetitive (CAR) domain that comprises a direct repetition of an 88 aa motif (residues 81–168 and 169–256) (31), which is poorly conserved even among the closely related magnetospirilla. Therefore, we confined our query to the N-terminal interaction domain (residues 23–81) plus the C-terminus (residues 293–426) of MamJ*_Mgryph_* (31). This generated a hit to the gene *mg-1g50* (locus tag: BEN30_00020). Similar to *mamJ* in the magnetospirilla, *mg-1g50* is located immediately upstream of *mamK*-II in the *mamDFHK*op in MV-1 (39). Despite only low overall sequence similarity to full-length MamJ_Mgryph_ (33%, 15% identity), Mg-1g50 shares 44% similarity (24% identity) and 43% similarity (18% identity) to the N- and C-termini of MamJ*_Mgryph_*, respectively, whereas it lacks the non-essential CAR domain and also differs considerably in size (Mg-1g50_MV-1_, 289 aa /30 kDa; MamJ*_Mgryph_*, 426 aa/44.3 kDa; Fig. S5). Moreover, the three-dimensional structures predicted by *Alphafold* for MamJ*_Mgryph_* (Fig. S5B) and Mg-1g50_MV-1_ (Fig. S5C) are distinct. A BLASTP search with full-length Mg-1g50_MV-1_ against the non-redundant database failed to reveal orthologues in any magnetic or non-magnetic bacterium.

We next transferred *mg-1g50* from MV-1 under the control of P*_mamDC_*_45_ together with *mamK*_MV-1_ into Δ*mamK_Mgryph_* and Δ*mamKY_Mgryph_* strains. Strikingly, the trans-complemented strain Δ*mamK_Mgryph_::mamK-mg-1g50*_MV-1_ now exhibited a *C*_mag_ close (97%) to the Δ*mamK_Mgryph_* strain complemented with native MamK*_Mgryph_* (Fig. 2D). The proportion of contiguous long MC raised to ~66%, which is also a significant increase compared to the mutant that was complemented with only *mamK*_MV-1_ (Δ*mamK_Mgryph_::mamK*_MV-1_, ~26%; Fig. 2C). Similarly, transfer of *mamK-mg-1g50*_MV-1_ in Δ*mamKY_Mgryph_* resulted in ~45% of long MCs (>10 particles) (Fig. 2C). Moreover, the occurrence of agglomerated magnetosomes or rings decreased from ~72% to ~37% compared to the parent Δ*mamKY_Mgryph_* strain (Fig. S4D). The *C*_mag_ was restored to 71% of the Δ*mamKY_Mgryph_::mamK_Mgryph_ C*_mag_, which is significantly higher than in the mutant trans-complemented with *mamK*_MV-1_ alone (19% of the Δ*mamKY_Mgryph_::mamK_Mgryph_ C*_mag_) (Fig. 2D). These results suggest that *Mg-1g50* indeed has a function similar to MamJ and helps to assemble magnetosomes efficiently into a MC even in the foreign host *Mgryph*.

### The resurrected MamK_LUCA_ MTB restores a non-fragmented MC

All MamK orthologs from diverse MTB showed partial to high complementation efficiency in *Mgryph*, suggesting that the function of the protein is evolutionary preserved. Therefore, we next asked whether a resurrected last common ancestor of MamK from the *Pseudomononadota* (formerly *Protoebacteria*), *Nitrospirota*, *Candidatus Omnitrophota*, and *Planctomycetota* phyla might still function as the extant MamK proteins in *Mgryph*. We used an approach of ancestral sequence reconstruction (ASR) to infer an ancient protein sequence on the basis of few extant ones. We attempted to reconstitute the MamK_LUCA_ sequence from diverse MTB using the maximum likelihood marginal reconstruction algorithm implemented in the Bio++ library (52, 53). The generated sequence termed MamK LUCA MTB (MamK_LUCA_) shares conserved residues of ATPase activity (Fig. S2), and the amino acid sequence shows 81% similarity and 51% identity to MamK from *Mgryph* (Table S1B). When the synthesized MamK_LUCA_ was fused to EGFP, it localized as a filament in *E. coli* like other MamK orthologs. However, unlike extant MamK orthologs, EGFP-MamK_LUCA_ localized homogenously distributed in the cytoplasma and often accumulated in patches in Δ*mamK_Mgryph_* (Fig. 3XI), indicating incomplete polymerization. Nevertheless, resurrected *mamK*_LUCA_ restored 52% of the *C*_mag_ of MamK*_Mgryph_* in Δ*mamK_Mgryph_* (Fig. 2D) and also restored mid-cell non-fragmented MC to a slightly higher frequency (~29%) than in Δ*mamK_Mgryph_* (~24%) (Fig. 2C). *MamK*_LUCA_ in Δ*mamKY_Mgryph_* recovered 24% *C*_mag_ of the Δ*mamKY_Mgryph_::mamK_Mgryph_ C*_mag_ (Fig. 2D), and the trans-complemented mutant showed a slightly higher frequency (10%) of MC with >10 particles (Δ*mamKY_Mgryph_*, ~4%; Fig. 2C). These results suggest that MamK_LUCA_ is functional in *Mgryph*, however, to a lower degree, possibly due to the lack of a hypothetical cognate ancestral MamJ-like adaptor protein.

### Mad28 is an actin-like protein with a MamK-like fold, partially restores MC formation in ΔmamK*_Mgryph_*, and forms filamentous structures *in vivo*

**Fig 4:**
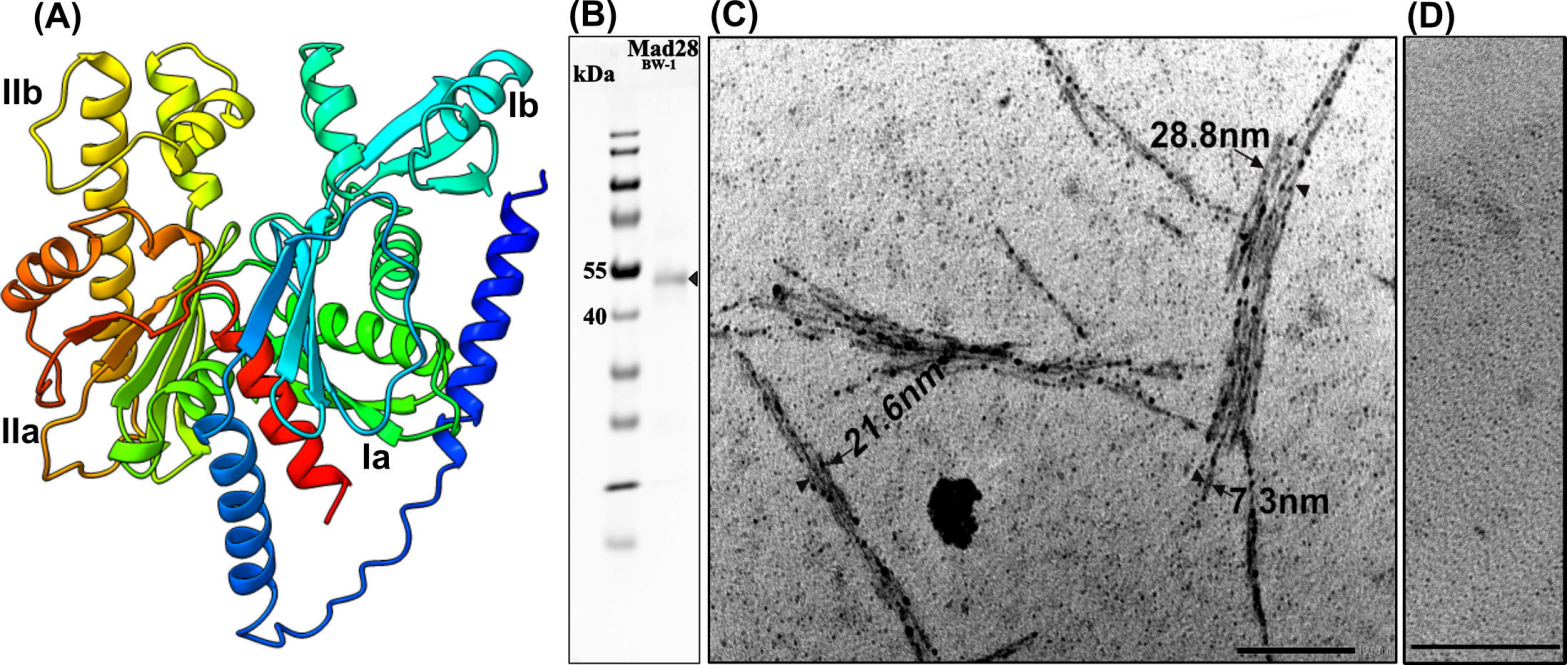
(**A**) Three-dimensional structure model of Mad28 from BW-1 predicted by *Alphafold* (https://www.rbvi.ucsf.edu/chimerax). The four actin domains, Ia, Ib, IIa, and IIb, are indicated in the structure. (**B**) SDS-PAGE of the purified N-terminal His-tagged Mad28_BW-1_ from *E. coli*. (**C**) *In vitro* polymerization of Mad28_BW-1_ in the presence of ATP-γ-S and without ATP (**D**) visualized by TEM. Scale bar, 100 nm.

We also studied the putative bacterial actin-like protein Mad28 (Fig. 1), which had been previously discovered in magnetotactic *Thermodesulfobacteriota* and *Nitrospirota* (38). To this end, we chose *mad28* genes from the thermodesulfobacterium BW-1 and *Nitrospirota* HCHbin1 and *Mbav* (with 15%–17% identity and 43%–45% similarity to MamK*_Mgryph_*). Alignment of MamK, Mad28, and MreB sequences revealed that three of the five conserved motifs involved in ATP binding (Fig. S2) are conserved in Mad28. These conserved motifs are a phosphate one binding site (xxxGxx), one adenosine-binding site (xxxxGGxx), and one connecting region 2 (Gx), indicating that Mad28 indeed might have evolved from an ATPase. Mad28 differs from MamK in the phosphate 2 binding site (MamK, xxxDxGxGxx; Mad28, xxxSxGxGxx) and the connecting region 1 (MamK, xEPx; Mad28, xExx), indicating that these motifs evolved differently and that the highly conserved aspartate (D) in phosphate 2 binding site evolved to serine (S). The three-dimensional structure of Mad28 from BW-1 modeled by *Alphafold* can be superimposed to Mad28 from *Nitrospirota* (HCH1 and Mbav), indicating almost identical structures (Fig. S6A). Remarkably, it also superimposes, if also not perfectly, with the actin-fold of MamK*_Mgryph_* (Fig. S6) with a conserved core structure that contains a nucleotide-binding and hydrolysis site (Fig. 4A).

We next tested whether Mad28 proteins can substitute MamK in *Mgryph*. Δ*mamK_Mgryph_* trans-complemented with *mad28* from BW-1, HCHbin1, and Mbav displayed a higher frequency of mid-cell non-fragmented MC (~38%, ~33%, and ~35%, respectively) than Δ*mamK_Mgryph_* (~24%; Fig. 2C), and the *C*_mag_ was about 86%–91% of the Δ*mamK_Mgryph_* mutant complemented with *mamK_Mgryph_* (Fig. 2D). In Δ*mamKY_Mgryph_*, *mad28* from these donors caused a slightly higher frequency of long chains (>10 particles, ~13%–19%) than in Δ*mamKY_Mgryph_* (~4%) (Fig. 2C), and the *C*_mag_ reached 16%–26% of the Δ*mamKY_Mgryph_::mamK_Mgryph_ C*_mag_ suggesting weak complementation. EGFP-Mad28 from BW-1 (*Thermodesulfobacteriota*), HCHbin1, and Mbav (*Nitrospirota*) showed a linear signal in *E. coli* (Fig. 3XII–xiv), indicating that Mad28 is capable to assemble into coherent filaments in *E. coli* like other actin-like proteins. In Δ*mamK_Mgryph_*, the localization pattern was essentially the same as in *E. coli* (Fig. 3XII–xiv).

### Mad28 interacts with MamK*_Mgryph_* and polymerizes *in vitro* in the presence of ATP-γ-S

As magnetotactic *Thermodesulfobacteriota* and *Nitrospirota* contain both MamK and Mad28 encoded in their genomes and because of the results above, the question was raised whether Mad28 and MamK interact and, possibly, co-assemble or form separate structures. We initially tested for co-localization of fluorescently tagged Mad28_BW-1_ and MamK*_Mgryph_ in vivo*. In *E. coli*, co-expressed EGFP-MamK*_Mgryph_* and mCherry-Mad28_BW-1_ formed superimposing filaments (Fig. S7A), suggesting that Mad28_BW-1_ and MamK*_Mgryph_* may interact with each other. A direct protein interaction between MamK*_Mgryph_* and Mad28_BW-1_ was probed by a bacterial adenylate cyclase two-hybrid assay (BACTH) (54). BACTH revealed a weak interaction between MamK*_Mgryph_* and Mad28_BW-1_ (Fig. S7B). However, the interaction was detected in only one of the tested permutations, indicating that the interaction between these proteins is either sterically constrained or limited due to strong competition between the interaction partners (i.e., self-interaction as well as possible putative interactions with other proteins). Mad28_BW-1_ showed no interaction with MamJ and MamY from *Mgryph* (Fig. S7B). To consolidate the interaction of Mad28 and MamK, we performed an immunoprecipitation assay. For this, we transferred *egfp-mad28*_BW-1_ and only the reporter gene *egfp* under the control of P*_mamDC_*_45_ into WT*_Mgryph_*. Subsequently, we extracted cell lysates from the modified strains and utilized GFP-trap agarose beads to capture both eGFP and eGFP-tagged Mad28 selectively from the cell lysates. Next, we performed Western blotting and immunodetection using antibodies against eGFP and MamK (Fig. S7Ci, Cii). Immunodetection of eGFP indicated the expression and successful pull down of eGFP and eGFP-Mad28. Interestingly, immunodetection of MamK showed a faint band in the eGFP-Mad28 expressing strain suggesting an interaction between Mad28 and native MamK*_Mgryph_* (Fig. S7Cii, right panel). These results suggest that Mad28 may also interact with native MamK orthologs of MTB belonging to *Thermodesulfobacteriota* and *Nitrospirota*.

We next asked whether Mad28 can polymerize *in vitro* in the presence of ATP-γ-S, which is a characteristic of other actin-like proteins. To this end, we expressed and purified Mad28_BW-1_ (44.55 kDa) from *E. coli* cells (Fig. 4B) and performed a polymerization assay, similar to that described before (55). TEM analysis of negatively stained Mad28_BW-1_ that had been incubated with ATP-γ-S confirmed that Mad28_BW-1_ indeed polymerized into filamentous structures (Fig. 4C), which were not detectable in the control in which ATP-γ-S was omitted (Fig. 4D). The well-developed bundles were >2 µm in length and ~29 nm in width, and the smallest bundles were ~7.3 nm in diameter. The dimensions and filamentous appearance of polymerized Mad28_BW-1_ are similar to those of the polymerized MamK from *Magnetospirillum* spp. (length, >2 µm; width, 6–7 nm) (21, 55) but distinct from the gently twisted double-helical filaments of ParM (14) and the straight or curved filamentous protofilament bundles or ring-like structures of MreB (56).

## DISCUSSION

The role of actin-like proteins in the organization and dynamics of MCs in MTB is still intriguing. *MamK* is the most highly conserved gene associated with magnetotaxis and present in all known MTB, which suggests that MamK is one of the primordial magnetosome proteins. However, experimental studies have been confined to MamK from the magnetotactic models *Mgryph* and AMB-1. Therefore, it is unknown whether the numerous insights derived from these related models can be generalized. We used the partial restoration of WT-like MC configuration and, specifically, the frequency of mid-cell non-fragmented MC in the MamK*_Mgryph_* mutant as measure to estimate the functionality of introduced foreign actin-like proteins, and the results were normalized to the strains that were complemented with the native *mamK_Mgryph_*. Expression of highly similar MamK from AMB-1 and the DCbin4 resulted in a substantially increased frequency of mid-cell non-fragmented MCs, which indicates a high degree of functionality for these proteins. In contrast, less similar MamK from MV-1, MC-1, RS-1, BW-1, Mbav, HCHbin1, and MYR-1 as well as MamK_LUCA_ also enhanced the frequency of mid-cell non-fragmented MC but to a lesser degree, which indicates limited functionality. These results were confirmed by another complementation experiment that was based on the ∆*mamKY_Mgryph_* mutant phenotype, where the frequency of long chains (>10 particles) was used as a measure for the functionality of the bacterial actin-like proteins. Here, we could include ParM from *E. coli*, a remote actin-like protein, which, however, was not able to complement. The only partial functionality of the mostly distantly related MamK in *Mgryph* could be due to several factors. While the ATPase domain of diverse MamKs is conserved, regions that mediate interaction with other proteins may have evolved divergently, resulting in limited functionality in *Mgryph*. Additionally, suboptimal expression levels may also contribute to the limited functionality. Because MamK alone is insufficient to assemble MCs, a further possible explanation for only partial complementation is the absence of cognate heterologous MamK interactors (such as MamJ) in *Mgryph*. We identified a putative *mamJ*-like gene (*mg-1g50*) in MV-1. Strikingly, co-expression of MamK and MamJ-like from MV-1 in ∆*mamK_Mgryph_* and ∆*mamKY_Mgryph_* significantly enhanced the degree of complementation. This suggests that MamJ-like MamK interactors may well exist in MTB outside the magnetotactic spirilla but simply have been overlooked so far. This indicates that sequence divergence is a determinative factor, likely in concert with the presence of a cognate MamK interaction partner with whom the protein has coevolved.

All tested MamK orthologs fused with EGFP localized as a filament in *E. coli*, indicating that these proteins share a core characteristic of actin-like proteins. These results show that the linear localization is independent of MTB-endogenous interaction partners and magnetosome membrane proteins, which is consistent with previous results observed with MamK from AMB-1 (15, 22). In contrast, these proteins showed different localization in ∆*mamK_Mgryph_*. MamK from *Alphaproteobacteria* (*Mgryph*, AMB-1, and MV-1) and *Candidatus* Etaproteobacteria (*Magnetococcales* MC-1 and Dcbin4) localized as a long filament in ∆*mamK_Mgryph_*, whereas those from *Thermodesulfobacteriota* (RS-1 and BW-1) and *Nitrospirota* (Mbav and HCHbin1) showed only short linear filaments. This might either be a typical property of these proteins or due to instability or rapid depolymerization of assembled filaments in *Mgryph* (i.e., altered subunit turnover or filament dynamics). EGFP-MamK from MYR-1, however, localized as a long filament indicating higher stability than other candidates from *Nitrospirota*. Interestingly, resurrected MamK_LUCA_ localized as a filament in *E. coli*, which indicated that the protein is able to polymerize. However, the localization in ∆*mamK_Mgryph_* was diffuse suggesting that the cellular environment including MamK-depolymerizing factors may play an essential role to form stable MamK filaments.

Mad28 from the thermodesulfobacterium BW-1 and the *Nitrospirota* HCHbin1 and Mbav also showed partial functionality in ∆*mamK_Mgryph_* and ∆*mamKY_Mgryph_* strains, as indicated by TEM. Furthermore, these Mad28 were found to localize similar to MamK in *E. coli* and ∆*mamK_Mgryph_* indicating that Mad28 is capable of directly assembling and localizing in a stable polymeric form. The lower degree of MC reconstitution detected by TEM correlated with lower *C*_mag_ values of complemented strains. The conserved ATPase residues and stable polymerization likely contribute to their partial restoration of MamK function in the deletion mutants, resulting in the reconstitution of magnetosome chains at variable degree. Our study provides the first evidence that Mad28 from BW-1 polymerized into stable filaments *in vitro*. The formation of filamentous structures reflects its specific and distinct intracellular physiological function in *Thermodesulfobacteriota* and *Nitrospirota* MTB. We also found that MamK and the putative actin-like Mad28 are able to self- and to cross-interact *in vivo* (based on bacterial two-hybrid experiments), that Mad28 forms filaments in *E. coli*, which co-localize with MamK filaments, and that they interact *in vitro* (based on immunoprecipitation assays). In magnetotactic *Thermodesulfobacteriota* and *Nitrospirota*, both genes co-occur, sometimes in several copies. The self- and cross-interacting nature plus interaction with putative MamJ-like proteins might account for the spectacular diversity of MC configurations and, in particular, for the presence of highly ordered multiple MCs in these uncultured bacteria. The Mad28 proteins from BW-1, Mbav, and HCH1 showed structural similarities indicating they may perform similar functions in these organisms. Moreover, even though Mad28 from BW-1 and MamK from *Mgryph* have low sequence identity/similarity (16%/45%), homology modeling suggests that they share structural similarities.

In conclusion, we provide the first comprehensive and systematic approach to test for conserved functionality of MamK and Mad28 proteins from diverse MTB in *Mgryph*. The study provides significant insights into the evolutionary relationships and functional conservation of bacterial actin-like proteins associated with magnetosomes. Furthermore, this study indicates the existence of species-specific MamK-interactors and expands the understanding of the molecular mechanisms underlying magnetosome chain organization in diverse MTB. It also establishes Mad28 as a novel bacterial actin likely to play a crucial role in the chain arrangement in MTB.

## MATERIALS AND METHODS

### Bacterial strains and culture conditions

*Mgryph* strains were cultivated under modified flask standard medium (FSM) (57) at 28°C and 120 rpm agitation in a micro-aerobic environment. *Escherichia coli* was cultured in lysogeny broth (LB) with shaking at 180 rpm at 37°C. The donor strain *E. coli* WM3064 (W. Metcalf, unpublished) was grown with 0.1 mM DL-a,Ɛ-diaminopimelic acid. Selection of transconjugants was carried out on agar-solidified media [1.5% (wt/vol)] by adding kanamycin at concentrations of 25 µg/mL (for *E. coli*) and 5 µg/mL (for *Mgryph*). Optical densities (OD) were measured photometrically at 565 nm for *Mgryph* strains and 600 nm for *E. coli*. The coefficient of magnetically induced differential light scattering (*C*_mag_, magnetic response) was determined as previously reported (58). The bacterial strains used in this study are listed in Table S1C.

### Molecular and genetic techniques

Oligonucleotides (Table S1D) were purchased from Sigma-Aldrich (Steinheim, Germany). Plasmids were constructed using standard recombinant techniques, as described below. All constructs were sequenced by Macrogen Europe (Amsterdam, Netherlands). The plasmids generated and utilized in this study are listed in Table S1E. The DNA synthesis of *mamK*s from MC-1, Dcbin4, Mbav, HCH-1, MYR-1, *mamK*_LUCA_, and *mad28*s from HCH-1 and Mbav was carried out by ATG:biosynthetics GmbH. Sequence-verified DNA fragments were delivered in a pGH standard vector with an ampR (bla) gene for selection on ampicillin. *MamK*s from MSR-1, AMB-1, MV-1, RS-1, and BW-1 and *mad28* from BW-1 were amplified by PCR from the respective genomic DNA.

### Phylogenetic tree reconstruction

A phylogenetic tree was built with bacterial actin-like proteins, including MamK, MreB, FtsA, and Mad28 of representative MTB. Sequences were aligned with the MAFFT software (59), then trimmed using the BMGE 1.12 (60) with low stringency parameters (-b 3 -g 0.5 options). The maximum-likelihood tree was built with the IQ-TREE v2.1.3 software (61) and a substitution model selected using ModelFinder (62) and the BIC. A set of 500 bootstrap replicates were conducted to calculate local support values. MreB was chosen as a root based on the evolutionary history of the actin ATPase protein family reconstructed by Ettema and colleagues (10).

### Construction of trans-complementation vectors

To trans-complement the ∆*mamK* and ∆*mamKY* strains of *Mgryph*, we cloned a fragment containing the respective foreign *mamK*/*mad28* gene and *parM* from *E. coli* into a Tn5-based insertion vector pBAM-Tn5-P*_mamDC_*_45_-*egfp*-HL with the constitutive promoter from *Mgryph*. To construct Tn5-based constructs containing *mamK*/*mad28*/*parM* gene without a reporter gene *egfp*, the plasmid pBAM-Tn5-P*_mamDC_*_45_-*egfp*-HL was digested with Ndel and EcoRV or Ndel and BamHI and ligated with amplified genes by PCR from respective genomic material digested with same restriction enzymes. To investigate the localization of MamK/Mad28 from distantly related donor strains and ParM from *E. coli*, we fused the genes with *egfp* and cloned them into the pBAM-Tn5-P*_mamDC_*_45_-*egfp*-HL. For fusion constructs, the plasmid pBAM-Tn5-P*_mamDC_*_45_-*egfp*-HL was digested with EcoRV and dephosphorylated using FastAP thermosensitive Alkaline Phosphatase (Thermo Scientific). The linearized plasmid was ligated with the genes amplified with phosphorylated primers. For the co-expression of *mamK* and *mv-1g50* from MV-1, these genes were placed under the control of P*_mamDC_* separated by an optimized ribosome binding site and cloned into pBAM-Tn5-P*_mamDC_*_45_-*egfp*-HL digested with Ndel and BamHI by Gibson assembly. The resulting constructs were transferred into *Mgryph* via conjugation, leading to the random insertion of the expression cassette into the host chromosome while keeping the foreign gene expression levels similar to that of the recipient.

### Construction of anhydrotetracycline-inducible expression vectors

For the construction of an anhydrotetracycline-inducible expression vector (pBAM-P*_tet_-egfp*), the fusion constructs of *mamK/mad28/parM* based on pBAM-Tn5-P*_mamDC_*_45_-*egfp* were digested with Ndel and BamHI or Ndel and XbaI and ligated into pBAM-P*_tet_-egfp* digested with same restriction enzymes.

### Conjugation and screening of transconjugants

Plasmid transfer by biparental conjugation was performed with donor strain *E. coli* WM3064 consisting of the verified construct and *Mgryph* strains as the acceptor strain as described previously (63). The generated trans-conjugant strains are listed in Table S1C. The transconjugants were transferred into 96-well plates with 150 µl of FSM containing the appropriate antibiotic concentration. The mutants were screened for integration of the expression cassette by PCR using primer pairs (Table S1D).

### Reconstruction of ancestral MamK

For the ASR of MamK protein, we first selected a set of 29 sequences from representative MTB strains or metagenome assembled genomes belonging to representative *Pseudomonadota*, *Thermodesulfobacteriota*, *Candidatus* Omnitrophota, *Planctomycetota*, and *Nitrospirota* phyla (see Fig. S1A for details). Sequences representing the major MTB phyla were selected using the public databases updated in 2019, while keeping a reasonable number of sequences to minimize the number of insertions/deletions. Indeed, actin proteins being highly divergent, their alignment generates many gaps, which is difficult to model in ASR. The multiple-sequence alignment was computed with MAFFT (59) using the default options. The gap positions of this alignment were then manually edited. The phylogenetic tree used for the ASR was computed using IQ-TREE (61). The amino acid substitution model used (LG + I + G_4_) was selected with the BIC. The tree was rooted using the clade containing the sequences from deep branching phyla including *Planctomycetota*, *Thermodesulfobacteriota*, and *Nitrospirota* phyla (Fig. S1).

For the ASR, we used the marginal maximum-likelihood reconstruction algorithm implemented in the Bio*++* software suite (53). Branch lengths were recomputed with Bio*++* using the branch and site heterogeneous substitution model COaLA (64). This substitution model uses the amino acid exchangeabilities from the LG model (65), and equilibrium frequencies are computed for the different branches of the tree using a correspondence analysis approach. Site heterogeneity is classically modeled using a gamma correction with four classes. The sequence chosen for experimental resurrection was the one located at the root of the tree. For each position in the sequence, we used the amino acid with maximal posterior probability.

### 3D structure prediction and superimpose

Prediction of the 3D structure of bacterial actin-like proteins was done using the Chimerax server (https://www.rbvi.ucsf.edu/chimerax). The amino acid sequences of the protein of interest were uploaded onto the Chimerax server, which automatically generated a 3D model through its built-in homology modeling algorithm. To validate the predicted structure, we employed Pymol (version 2.5.0) to superimpose the predicted models of actin-like proteins to compare the models.

### Fluorescence microscopy

To study the localization of EGFP fusion proteins, three-dimensional structured illumination fluorescence microscopy was conducted using an Eclipse Ti2-E N-SIM E fluorescence microscope (Nikon) equipped with a CFI SR Apo TIRF AC 100× H NA1.49 oil objective lens, a hardware-based “perfect focus system,” and an Orca Flash4.0 LT Plus sCMOS camera (Hamamatsu). Sample preparation, fluorescence excitation with the 488-nm laser line for imaging GFP, and image reconstruction and analysis were carried out as previously described (66).

### Protein expression and purification

Recombinant protein expression was carried out in *E. coli* BL21 (DE3) grown in LB medium supplemented with 50 µg/mL kanamycin. The *E. coli* cells were transformed with a plasmid (pET28a-Mad28_BW-1_) encoding Mad28_BW-1_ fused to a 6xHis tag. The plasmid (pET28a-Mad28_BW-1_) was constructed by restriction and ligation where amplified *mad28*_BW-1_ was cloned into pET28a digested with Ndel and BamHI. The culture was inoculated with 1/10 volume of a fully grown overnight culture and cultivated at 37°C until the OD_600_ reached 0.6–0.9. The temperature was then reduced to 16°C, and after 30 min, 0.5 mM isopropyl-β-D-thiogalactopyranoside (IPTG) was added to induce gene expression. After 16 hours of induction, the cells were harvested by centrifugation at 10,000 rpm for 10 minutes at 4°C and resuspended in lysis buffer [25 mM HEPES (pH 7.8), 500 mM NaCl, 10 mM imidazole, 10% glycerol, 0.1% Triton-X-100]. The cells were incubated on ice for 30 minutes, followed by lysing using a Microfluidizer (microfluidics) by passage at 1,500 bar at 4°C. The crude extract was then centrifuged at 10,000 rpm for 20 minutes at 4°C, and the resulting supernatant was treated as the soluble protein fraction.

The lysate was loaded onto a Ni-NTA resin column (Jena Bioscience) pre-equilibrated with lysis buffer. The column was washed with 10 column volumes of wash buffer [25 mM HEPES (pH 7.8), 500 mM NaCl, 20 mM imidazole, 10% glycerol, 0.1% Triton-X-100] to remove nonspecifically bound proteins. The Mad28_BW-1_ was eluted with an elution buffer containing 500 mM imidazole. Protein concentration was determined using the Bradford assay (67), and purity was assessed by SDS-PAGE (68). The purified protein was dialyzed against a storage buffer [25 mM HEPES (pH 8.0), 100 mM NaCl, 0.2 mM EDTA, and 20% glycerol] and stored at −80°C until further use.

### Polymerization assay

The purified Mad28_BW-1_ was assayed for *in vitro* polymerization using a protocol adapted from reference (55). A total volume of 30 µL of polymerization buffer, containing 50 mM Tris (pH 7.0), 100 mM NaCl, 14 mM MgCl2, and 30 mM KCl, was used. Purified Mad28_BW-1_ protein was added at a final concentration of 10 µM, and the mixture was centrifuged at 15,000 rpm and 4°C for 30 minutes using a Centrifuge 5424R to remove aggregates. Next, a non-hydrolyzable ATP analog, adenosine-5′-(γ-thio) triphosphate (ATP-γ-S), was added at a final concentration of 2 mM, and the samples were incubated for 15 minutes at 30°C. A carbon-coated copper grid was then placed on a drop of the mixture for 6 minutes and washed with ddH_2_O. The grid was negatively stained with 2% (wt/vol) uranyl acetate for 1 minute and observed using a JEM-2100 transmission electron microscope at 80 kV.

### Bacterial two-hybrid assay

To conduct protein interaction studies using the adenylate cyclase (CyaA)-based two-hybrid assay (54), we amplified *mad28*_BW-1_ from genomic DNA of *D. magnetovallimortis* and cloned into pUT18C, pUT18, pKT25, and pKNT25 plasmids using primers (listed in Table S1C) that contained suitable restriction endonuclease sites for cloning into the two-hybrid variant vectors. The resulting T18- and T25-based plasmids were confirmed by DNA sequencing and co-transformed into the *E. coli* BTH101 reporter strain. To assess the interaction between the proteins, cells were plated on LB agar supplemented with 40 µg/mL 5-bromo-4-chloro-3-indolyl-β-D-galactopyranoside (X-Gal), 0.5 mM IPTG, ampicillin (100 µg/mL), and kanamycin (50 µg/mL) and incubated at 28°C. Colonies were subsequently analyzed for the blue color formation. To visualize the outcome of the experiments, several colonies per plasmid combination were grown overnight at 28°C in LB liquid medium containing IPTG (0.5 mM), ampicillin (100 µg/mL), and kanamycin (50 µg/mL), and 3 µL of culture was spotted onto M63 mineral salts agar supplemented with 0.2% (wt/vol) maltose, X-Gal (40 µg/mL), 0.5 mM IPTG, ampicillin (50 µg/mL), and kanamycin (25 µg/mL). The M63 plates were incubated at 28°C for 2 days and documented with a camera (Panasonic). The blue coloration was regarded as positive. Constructs carrying a leucine zipper fused with the T18- and T25-fragment were used as positive controls. Co-transformants harboring constructs coding for the respective T18- and T25- protein fusions in combination with the corresponding T25- and T18- subunit alone served as negative controls.

### Immunoprecipitation and immunoblotting

To immunoprecipitate GFP-tagged Mad28_BW-1_, GFP-Trap magnetic agarose beads from Chromotek were used following the manufacturer’s protocol. The supernatant of cell lysate of *Mgryph* strains expressing eGFP-Mad28_BW-1_ was incubated with the beads for 2 hours at 4°C with gentle shaking. After incubation, the beads were washed five times with wash buffer, and Mad28_BW-1_ was eluted by heating the beads in SDS sample buffer (58 mM Tris–HCl, pH 6.8, 2% SDS, 5% glycerol, 0.1 M DTT, 0.01% bromophenol blue) at 95°C for 10 minutes. The proteins were separated by SDS-PAGE using 12% gels and Tris-glycine running buffer (50 mM Tris–HCl, pH 8.5, 0.19 M glycine, 0.1% SDS), as described in reference (69).

To transfer the proteins onto PVDF membranes, the semi-dry technique was used at 2 mA cm^−2^ for 2 hours, with Bjerrum–Schafer-Nielsen transfer buffer (48 mM Tris-base, 39 mM glycine, 0.0375% SDS, 20% methanol) (70). The membrane was blocked with 5% non-fat dry milk in Tris-buffered saline containing 0.1% Tween-20 for 1 hour at room temperature and incubated with the primary antibody against the protein of interest overnight at 4°C. Afterward, membranes were washed four times with Tween-Tris-buffered saline (TTBS) buffer (0.05% Tween 20, 50 mM Tris–HCl pH 7.5, 150 mM NaCl) and incubated with HRP-labeled anti-rabbit secondary antibodies for 1 hour, followed by further washing with TTBS. Immunodetection was performed using the commercial Western Blot Chemiluminescence HRP Substrate by Takara Bio (USA). Finally, images of the gel and blot were captured with a ChemiDoc XRS+ System (Bio-Rad, USA) and processed with ImageLab 6.0.1 software.

### Transmission electron microscopy

For TEM analysis, the strains were cultivated anaerobically in FSM at 24°C for 48 hours, fixed in 1.8% formaldehyde, and adsorbed onto carbon-coated copper grids (F200-CU carbon support film, 200 mesh; Electron Microscopy Sciences, Hatfield, UK). The samples were then washed three times with double-distilled water. TEM analysis was performed using a JEM-2100 instrument (JEOL, Ltd., Tokyo, Japan) at 80 kV, and images were captured with a Gatan model 782 ES500W Erlangshen CCD camera (Gatan, Inc., Pleasanton, CA) and Digital Micrograph 1.80.70 software (Gatan, Inc.). ImageJ Fiji V1.50c (71) software was used for data analysis and measurements. The statistical analysis of magnetosome organization in the mutants was performed using GraphPad Prism statistical software.

## SUPPLEMENTAL MATERIAL

Fig. S1 mbio.01649-23-s0001.tif The maximum-likelihood tree used as a guide to perform the ancestral sequence reconstruction of MamKLUCA.

Fig. S2 mbio.01649-23-s0002.tif The alignments of MamK, Mad28, and MreB protein sequences.

Fig. S3 mbio.01649-23-s0003.tif Magnetic response (Cmag) of transcomplemented mutants.

Fig. S4 mbio.01649-23-s0004.tif Representative TEM micrographs of cells displaying chain localization variants and analysis of cells displaying the magnetosome organization in cell population.

Fig. S5 mbio.01649-23-s0005.tif Alignment of protein sequences of Mg-1g50MV-1 and MamJMgryph.

Fig. S6 mbio.01649-23-s0006.tif Three-dimensional (3D) structure model alignment of Mad28 proteins.

Fig. S7 mbio.01649-23-s0007.tif Co-expression and localization, BACTH analysis, and immunoprecipitation assay.

Table S1 mbio.01649-23-s0008.docx Protein sequences used for LUCA reconstruction, percentage identities/similarities, strains, primers, and plasmids used in this study.

ASM does not own the copyrights to Supplemental Material that may be linked to, or accessed through, an article. The authors have granted ASM a non-exclusive, world-wide license to publish the Supplemental Material files. Please contact the corresponding author directly for reuse.
