# Supplementary figures and images for: Experimental analysis of diverse actin-like proteins from various magnetotactic bacteria by functional expression in Magnetospirillum gryphiswaldense
Source: mBio. 2023 Oct 12;14(5):e01649-23. doi: 10.1128/mbio.01649-23 (PMC10653835; doi:10.1128/mbio.01649-23)

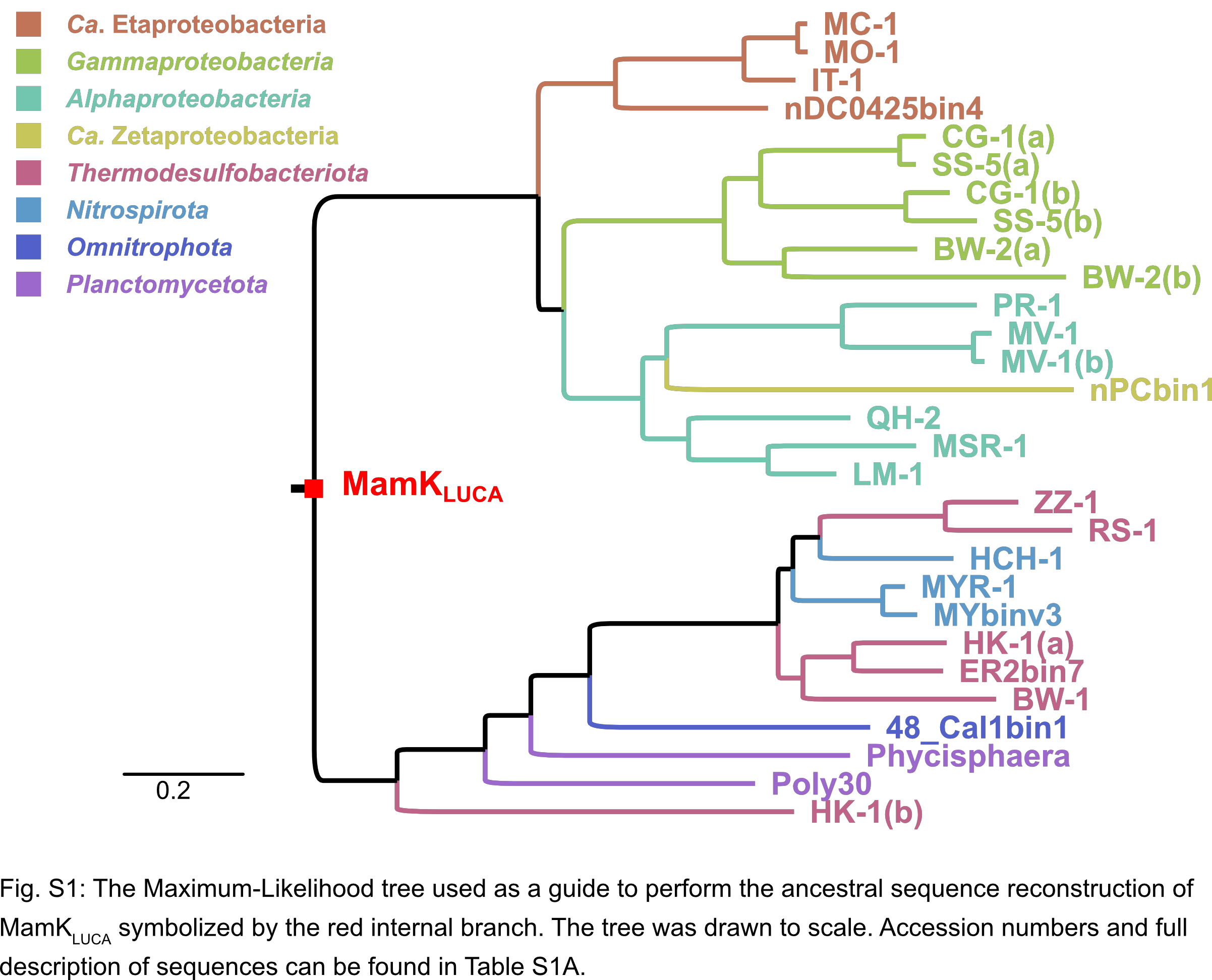

Supplement: Fig. S1 — The maximum-likelihood tree used as a guide to perform the ancestral sequence reconstruction of MamKLUCA. [file mbio.01649-23-s0001.tif]

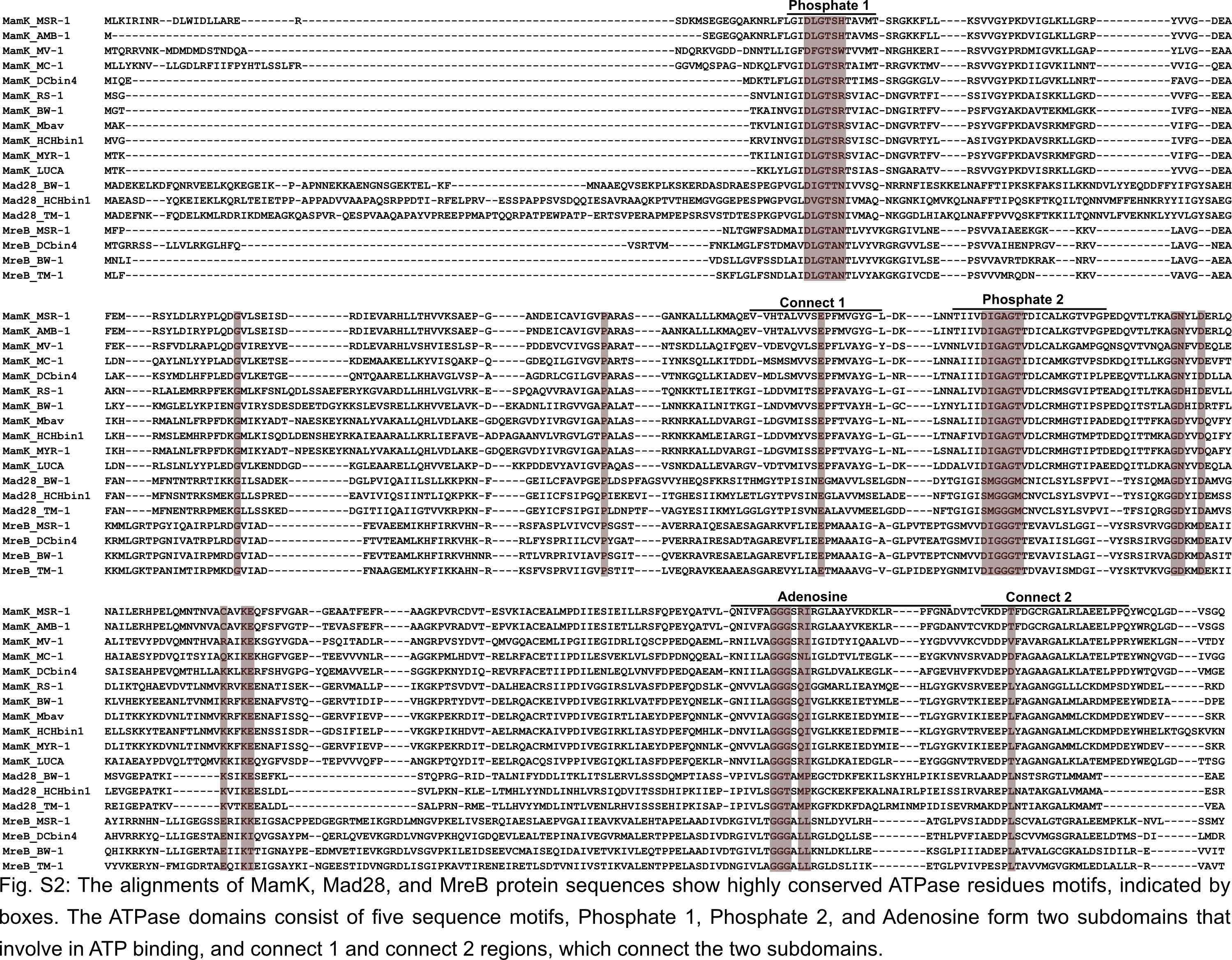

Supplement: Fig. S2 — The alignments of MamK, Mad28, and MreB protein sequences. [file mbio.01649-23-s0002.tif]

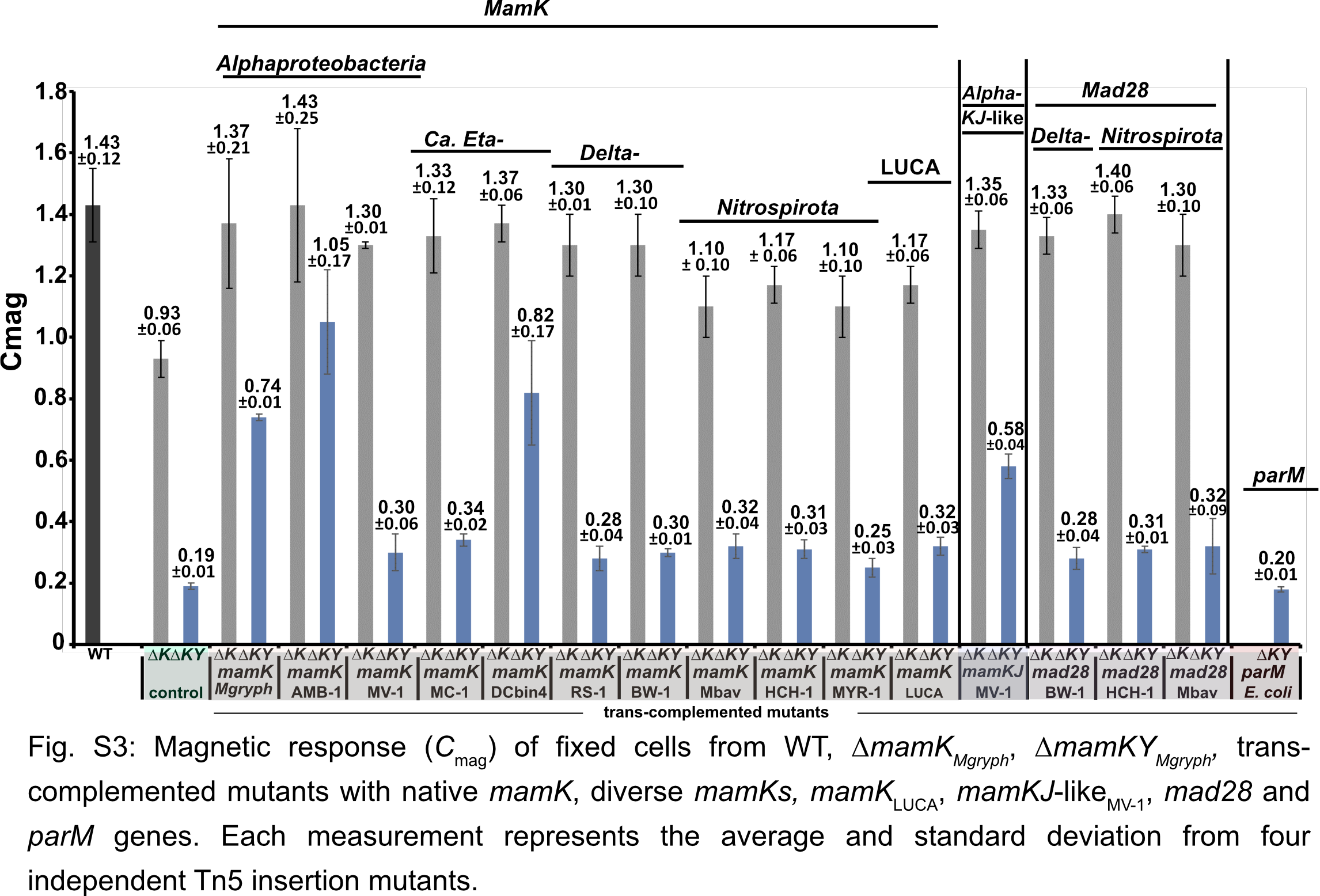

Supplement: Fig. S3 — Magnetic response (Cmag) of transcomplemented mutants. [file mbio.01649-23-s0003.tif]

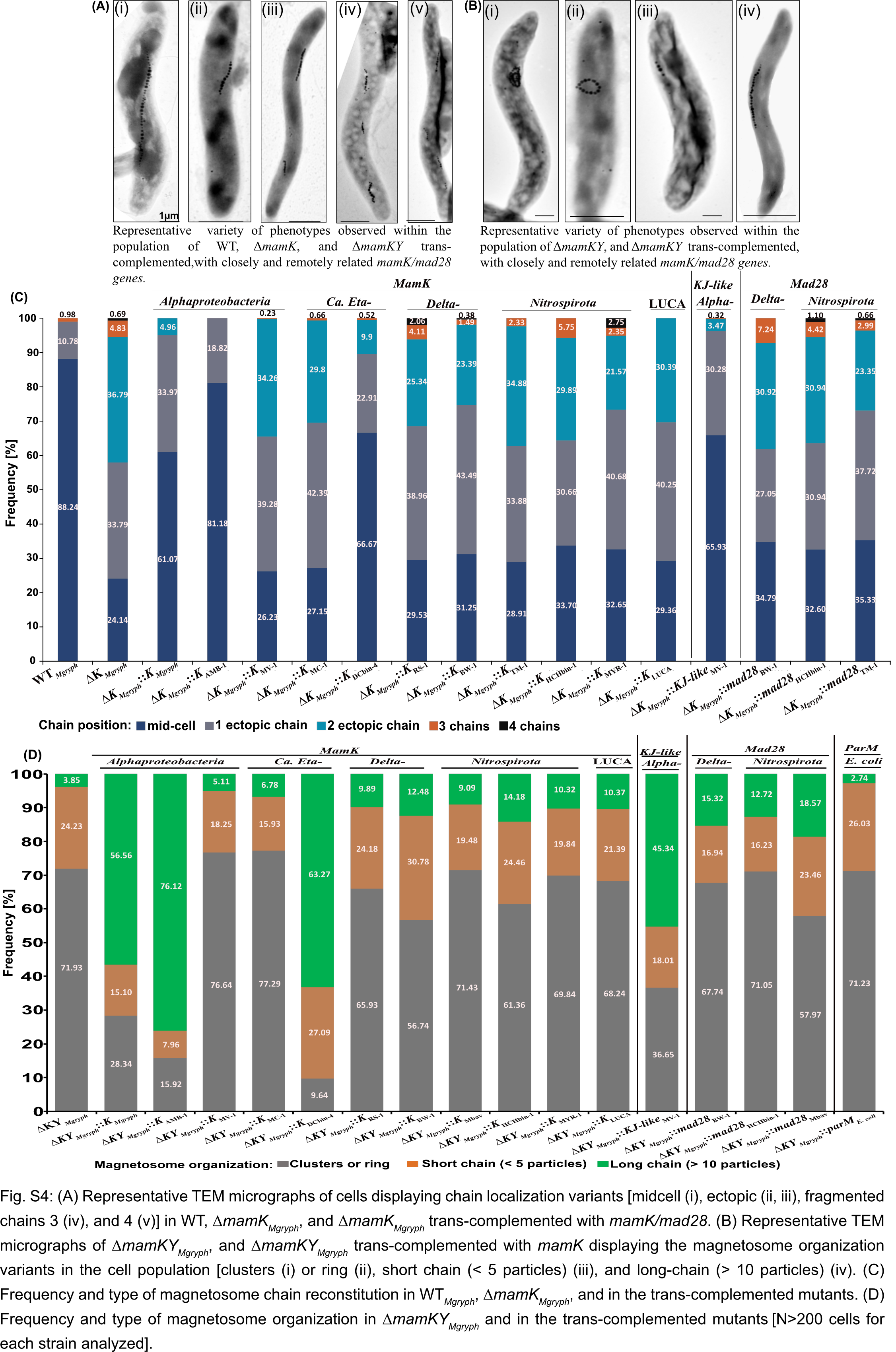

Supplement: Fig. S4 — Representative TEM micrographs of cells displaying chain localization variants and analysis of cells displaying the magnetosome organization in cell population. [file mbio.01649-23-s0004.tif]

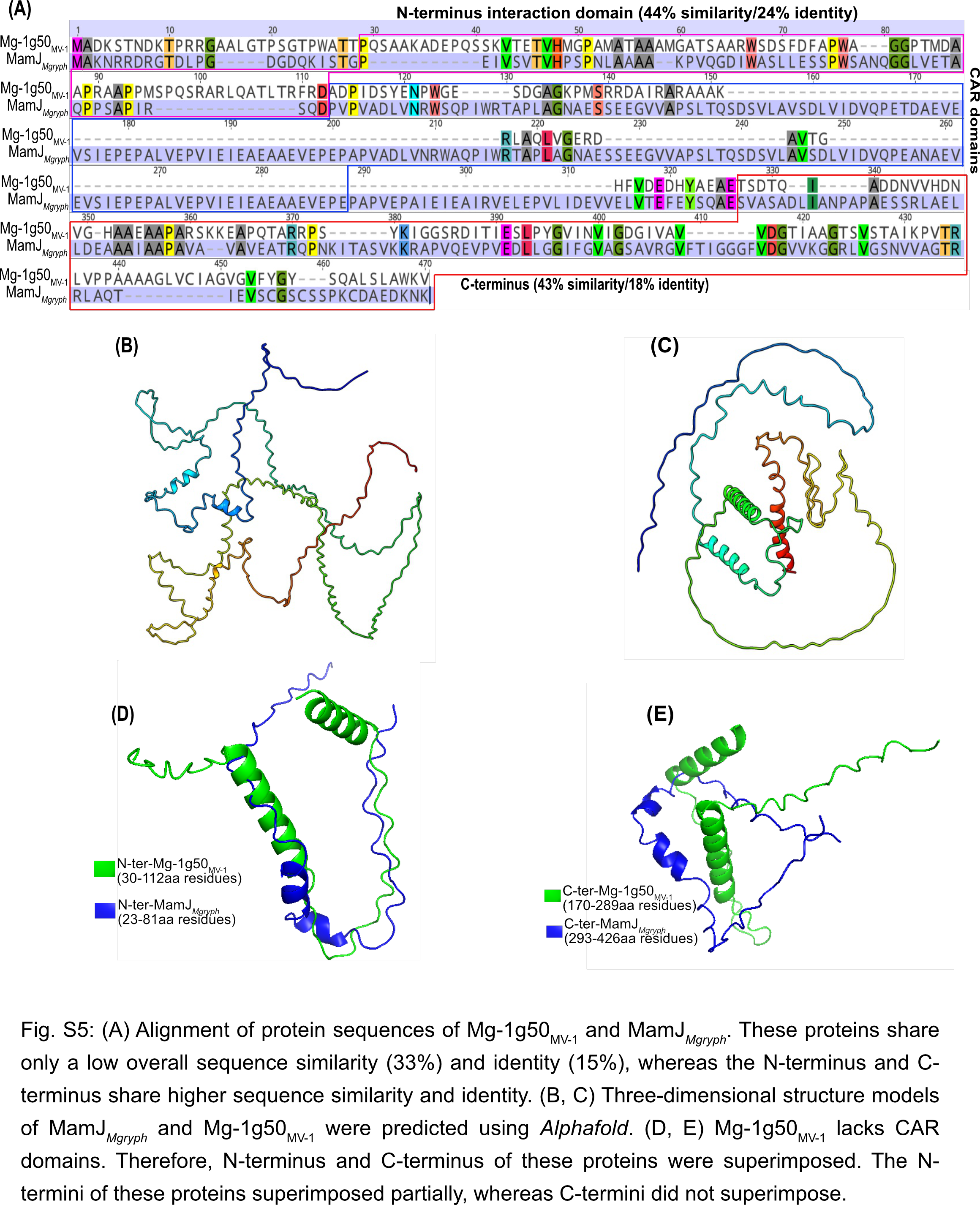

Supplement: Fig. S5 — Alignment of protein sequences of Mg-1g50MV-1 and MamJMgryph. [file mbio.01649-23-s0005.tif]

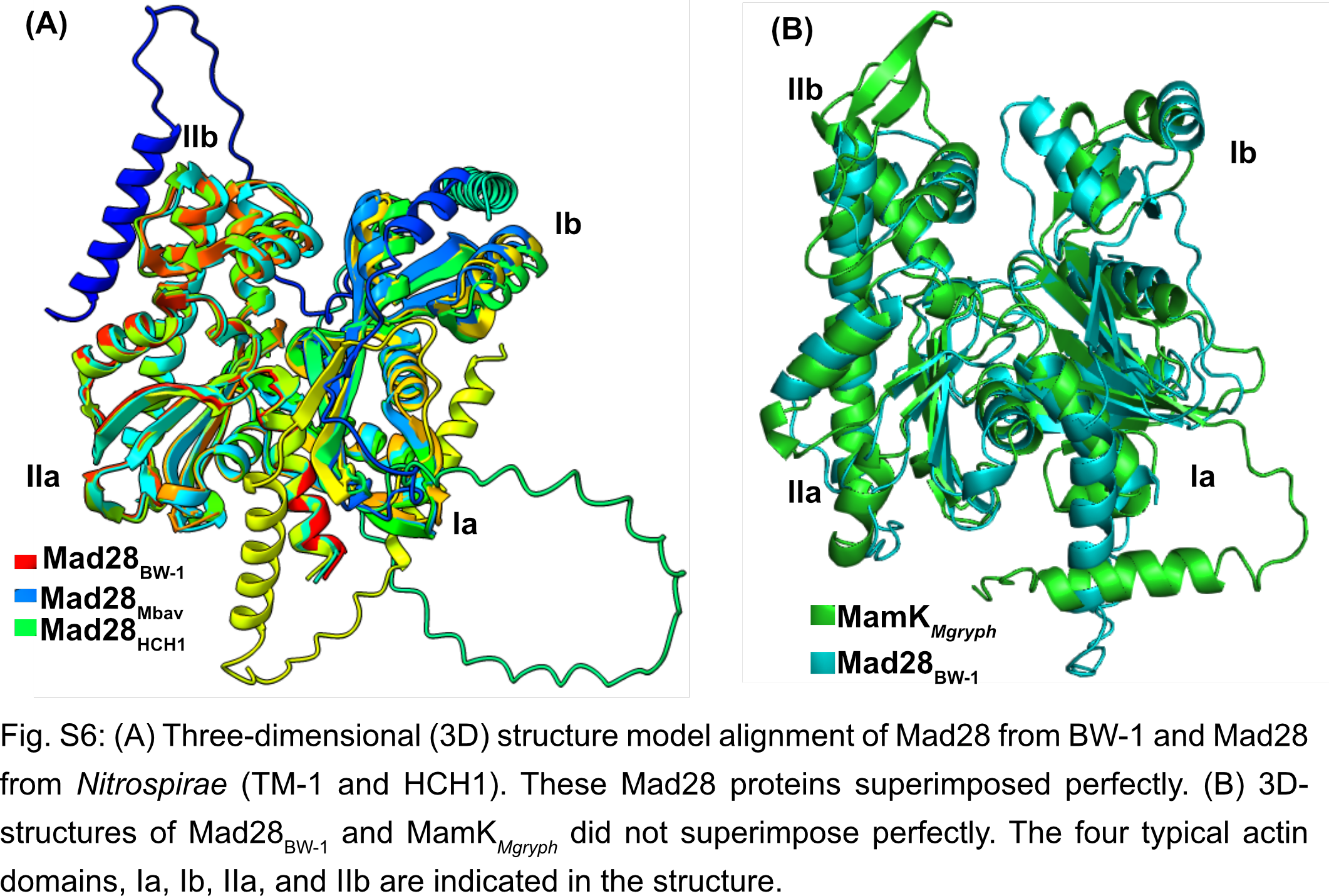

Supplement: Fig. S6 — Three-dimensional (3D) structure model alignment of Mad28 proteins. [file mbio.01649-23-s0006.tif]

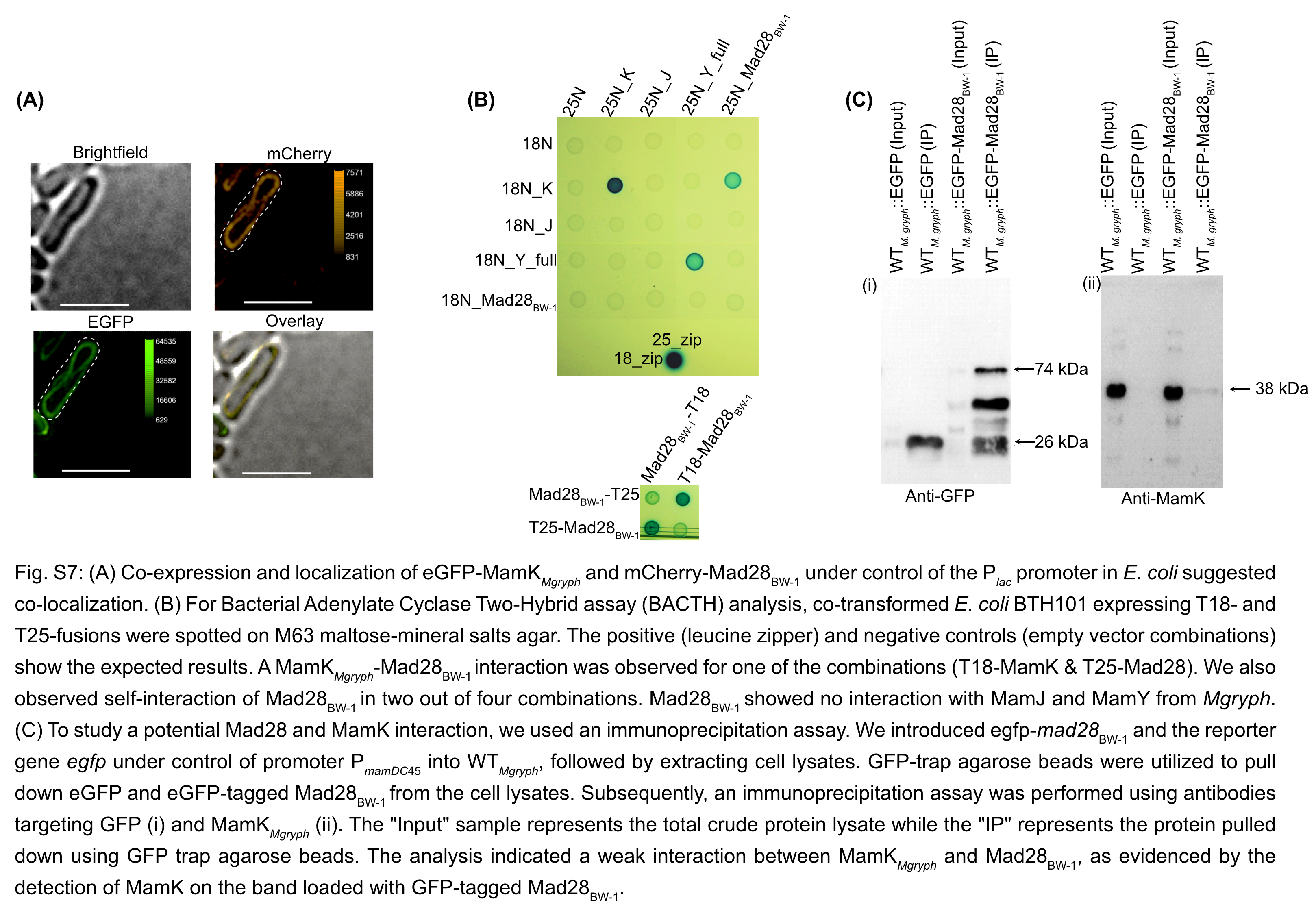

Supplement: Fig. S7 — Co-expression and localization, BACTH analysis, and immunoprecipitation assay. [file mbio.01649-23-s0007.tif]
