# Supplementary material for: Experimental analysis of diverse actin-like proteins from various magnetotactic bacteria by functional expression in Magnetospirillum gryphiswaldense
Source: mBio. 2023 Oct 12;14(5):e01649-23. doi: 10.1128/mbio.01649-23 (PMC10653835; doi:10.1128/mbio.01649-23)
Supplement: Table S1 — Protein sequences used for LUCA reconstruction, percentage identities/similarities, strains, primers, and plasmids used in this study. [file mbio.01649-23-s0008.docx]

| **Table S1A:** List of protein sequences used for the ancestral sequence reconstruction of MamK LUCA. | | | | | |
| --- | --- | --- | --- | --- | --- |
| Taxonomy | |  | Name of the strain/isolate | Accession number NCBI | reference |
| Phyla | Class |  |  |  |  |
|  |  |  |  |  |  |
| *Pseudomonadota* |  |  |  |  |  |
| **.** | *Alphaproteobacteria* |  |  |  |  |
|  |  | **.** | *Candidatus* Magneticavibrio boulderlitore LM-1 | CAA6606531.1 | (1) |
|  |  | **.** | *Magnetospirillum gryphiswaldense* MSR-1 | WP_024080588.1 | (2) |
|  |  | **.** | *Magnetospira thiophila* QH-2 | WP_252508680.1 | (3) |
|  |  | **.** | *Magnetovibrio blakemorei* MV-1 | (a) WP_245648941.1 | (4) |
|  |  |  |  | (b) WP_084005269.1 | (4) |
|  |  | **.** | *Candidatus* Terasakiella magnetica PR-1 | CAA7623094.1 | (1) |
| **.** | *Candidatus* Etaproteobacteria |  |  |  |  |
|  |  | **.** | Magnetococcales bacterium nDC0425bin4 | MBF0623191.1 | (5) |
|  |  | **.** | *Magnetofaba australis* IT-1 | WP_085440046.1 | (6) |
|  |  | **.** | *Candidatus* Magnetococcus massalia MO-1 | CRH04720.1 | (7) |
|  |  | **.** | Magnetococcus marinus MC-1 | WP_011713881.1 | (8) |
| . | *Gammaproteobacteria* |  |  |  |  |
|  |  | **.** | Ectothiorhodospiraceae sp. BW-2 | (a) QEP43995.1 | (9) |
|  |  |  |  | (b) QEP43992.1 | (9) |
|  |  | **.** | *Chromatiaceae* sp. SS-5 | (a) AFX88989.1 | (10) |
|  |  |  |  | (b) AFX88988.1 | (10) |
|  |  | **.** | *Chromatiaceae* sp. CG-1 | (a) MTBCG1_v1_60137* | (11) |
|  |  |  |  | (b) MTBCG1_v1_60139* | (11) |
| . | *Candidatus* Zetaproteobacteria |  |  |  |  |
|  |  |  | Candidatus Zetaproteobacteria nPCbin1 | MBF0281874.1 | (5) |
| *Thermodesulfobacteriota* |  |  |  |  |  |
|  |  | **.** | *Desulfamplus magnetovallimortis* BW-1 | WP_080798239.1 | (12) |
|  |  | **.** | *Solidesulfovibrio magneticus* RS-1 | WP_015862714.1 | (13) |
|  |  | **.** | *Candidatus* Magnetomorum sp. HK-1 | (a) KPA14283.1 |  |
|  |  |  |  | (b) KPA14705.1 | (14) |
|  |  | **.** | *Deltaproteobacteria* bacterium ER2bin7 | MBF0450772.1 | (5) |
|  |  | **.** | *Deltaproteobacteria* sp. ZZ-1 | MTBZZ1_v1_170065* | (15) |
| *Nitrospirota* |  |  |  |  |  |
|  |  |  | Nitrospirota bacterium MYbinv3 | GCA_002753395.1 | (5) |
|  |  |  | *Candidatus* Magnetobacterium casensis MYR-1 | WP_040335344.1 | (16) |
|  |  |  | *Candidatus* Magnetominusculus xianensis HCH-1 | KWT94826.1 | (17) |
| *Planctomycetota* |  |  |  |  |  |
|  |  |  | *Phycisphaera mikurensis* | WP_014436216.1 | (18) |
|  |  |  | *Planctomycetes* sp. Poly30 | QDV07157.1 | (19) |
| *Omnitrophota* |  |  |  |  |  |
|  |  |  | *Omnitrophota* sp. Calbin1 | MBF0594750.1 | (5) |
|  |  |  |  |  |  |
| *Data are publically available on the Microscope Plateform through https://mage.genoscope.cns.fr | | | | | |

**Table S1B**: Percentage identities/similarities of actin-like proteins of MamK from *Mgryph*.

|  | **%**  **Identity** | **%**  **Similarity** |
| --- | --- | --- |
| **MamK** |  |  |
| AMB-1 | 95 | 98 |
| MV-1 | 50 | 79 |
| MC-1 | 47 | 79 |
| DCbin4 | 53 | 83 |
| RS-1 | 38 | 71 |
| BW-1 | 37 | 71 |
| Mbav | 35 | 72 |
| HCHbin1 | 38 | 71 |
| MYR-1 | 35 | 72 |
|  |  |  |
| **LUCA** | 51 | 81 |
|  |  |  |
| **Mad28** |  |  |
| BW-1 | 16 | 45 |
| HCHbin1 | 15 | 43 |
| Mbav | 17 | 43 |
|  | **MamK**  **Mgryph** | **MamK**  **Mgryph** |

**Table S1C:** Strains used in this study.

| Bacterial strains | Characteristics | References |
| --- | --- | --- |
| Strains  *E. coli* |  |  |
| *E. coli* Neb10ß | F- *mcrA* Δ(*mrr*-*hsd-*RMS*mcr*BC)  Φ80d*lac*ZΔM15  Δ*lac*X74 *end*A1 *rec*A1 *deo*R  Δ(*ara*,*leu*)7697 *ara*D139 *gal*  U *gal*K *nup*G *rps*L λ- | Purchased from NEB BioLabs Inc (MA, USA) |
| *E. coli* WM3064  *M. gryphiswaldense* | *thrB1004 pro thi rpsL hsdS lacZ∆M15 RP4-1360 ∆(araBAD)567 ∆dapA1341::[erm pir]* | William Metcalf, Universität von Illinois (UIUC), USA, unpublished |
| Wild type | Lab strain-Wildtype, *Rif^R^, Sm^R^* | (20) |
| ∆*mamK_Mgryph_* | deletion of *mamK* | (21) |
| ∆*mamKY_Mgryph_* | deletion of *mamK*, *mamY* | (22) |
| ∆*mamK_Mgryph_*::*mamK_Mgryph_* | Strain with chromosomally inserted *mamK_Mgryph_* in ∆*mamK_Mgryph_* | This study |
| ∆*mamK_Mgryph_*::*mamK*_AMB-1_ | Strain with chromosomally inserted *mamK*_AMB-1_ in ∆*mamK_Mgryph_* | This study |
| ∆*mamK_Mgryph_*::*mamK*_MV-1_ | Strain with chromosomally inserted *mamK*_MV-1_ in ∆*mamK_Mgryph_* | This study |
| ∆*mamK_Mgryph_*::*mamK*_MC-1_ | Strain with chromosomally inserted *mamK*_MC-1_ in ∆*mamK_Mgryph_* | This study |
| ∆*mamK_Mgryph_*::*mamK*_DCbin4_ | Strain with chromosomally inserted *mamK*_DCbin4_ in ∆*mamK_Mgryph_* | This study |
| ∆*mamK_Mgryph_*::*mamK*_RS-1_ | Strain with chromosomally inserted *mamK*_RS-1_ in ∆*mamK_Mgryph_* | This study |
| ∆*mamK_Mgryph_*::*mamK*_BW-1_ | Strain with chromosomally inserted *mamK*_BW-1_ in ∆*mamK_Mgryph_* | This study |
| ∆*mamK_Mgryph_*::*mamK*_TM-1_ | Strain with chromosomally inserted *mamK*_TM-1_ in ∆*mamK_Mgryph_* | This study |
| ∆*mamK_Mgryph_*::*mamK*_HCH-1_ | Strain with chromosomally inserted *mamK*_HCH-1_ in ∆*mamK_Mgryph_* | This study |
|  |  |  |
| ∆*mamK_Mgryph_*::*mamK*_MYR-1_ | Strain with chromosomally inserted *mamK*_MYR-1_ in ∆*mamK_Mgryph_* | This study |
| ∆*mamK_Mgryph_*::*mamK*_LUCA_ | Strain with chromosomally inserted *mamK*_LUCA_ in ∆*mamK_Mgryph_* | This study |
| ∆*mamK_Mgryph_*::*mad28*_BW-1_ | Strain with chromosomally inserted *mad28*_BW-1_ in ∆*mamK_Mgryph_* | This study |
| ∆*mamK_Mgryph_*::*mad28*_HCH-1_ | Strain with chromosomally inserted *mad28*_HCH-1_ in ∆*mamK_Mgryph_* | This study |
| ∆*mamK_Mgryph_*::*mamK*_TM-1_ | Strain with chromosomally inserted *mamK*_TM-1_ in ∆*mamK_Mgryph_* | This study |
| ∆*mamKY_Mgryph_*::*mamK_Mgryph_* | Strain with chromosomally inserted *mamK_Mgryph_* in ∆*mamKY_Mgryph_* | This study |
| ∆*mamKY_Mgryph_*::*mamK*_AMB-1_ | Strain with chromosomally inserted *mamK*_AMB-1_ in ∆*mamKY_Mgryph_* | This study |
| ∆*mamKY_Mgryph_*::*mamK*_MV-1_ | Strain with chromosomally inserted *mamK*_MV-1_ in ∆*mamKY_Mgryph_* | This study |
| ∆*mamKY_Mgryph_*::*mamK*_MC-1_ | Strain with chromosomally inserted *mamK*_MC-1_ in ∆*mamKY_Mgryph_* | This study |
| ∆*mamKY_Mgryph_*::*mamK*_DCbin4_ | Strain with chromosomally inserted *mamK*_DCbin4_ in ∆*mamKY_Mgryph_* | This study |
| ∆*mamKY_Mgryph_*::*mamK*_RS-1_ | Strain with chromosomally inserted *mamK*_RS-1_ in ∆*mamKY_Mgryph_* | This study |
| ∆*mamKY_Mgryph_*_::_*mamK*_BW-1_ | Strain with chromosomally inserted *mamK*_BW-1_ in ∆*mamKY_Mgryph_* | This study |
| ∆*mamKY_Mgryph_*_::_*mamK*_TM-1_ | Strain with chromosomally inserted *mamK*_TM-1_ in ∆*mamKY_Mgryph_* | This study |
| ∆*mamKY_Mgryph_*::*mamK*_HCH-1_ | Strain with chromosomally inserted *mamK*_HCH-1_ in ∆*mamKY_Mgryph_* | This study |
| ∆*mamKY_Mgryph_*::*mamK*_MYR-1_ | Strain with chromosomally inserted *mamK*_MYR-1_ in ∆*mamKY_Mgryph_* | This study |
| ∆*mamKY_Mgryph_*::*mamK*_LUCA_ | Strain with chromosomally inserted *mamK*_LUCA_ in ∆*mamKY_Mgryph_* | This study |
| ∆*mamKY_Mgryph_*::*mad28*_BW-1_ | Strain with chromosomally inserted *mad28*_BW-1_ in ∆*mamKY_Mgryph_* | This study |
| ∆*mamKY_Mgryph_*::*mad28*_HCH-1_ | Strain with chromosomally inserted *mad28*_HCH-1_ in ∆*mamKY_Mgryph_* | This study |
| ∆*mamKY_Mgryph_*::*mad28*_TM-1_ | Strain with chromosomally inserted *mad28*_TM-1_ in ∆*mamKY_Mgryph_* | This study |
| ∆*mamK_Mgryph_*::*egfp-mamK_Mgryph_* | Strain with chromosomally encoded EGFP-MamK*_Mgryph_* fusion expressed in ∆*mamK_Mgryph_* | This study |
| ∆*mamK_Mgryph_*::*egfp-mamK*_AMB-1_ | Strain with chromosomally encoded EGFP-MamK_AMB-1_fusion expressed in ∆*mamK_Mgryph_* | This study |
| ∆*mamK_Mgryph_*::*egfp-mamK*_MV-1_ | Strain with chromosomally encoded EGFP-MamK_MV-1_fusion expressed in ∆*mamK_Mgryph_* | This study |
| ∆*mamK_Mgryph_*::*egfp-mamK*_MC-1_ | Strain with chromosomally encoded EGFP-MamK_MC-1_fusion expressed in ∆*mamK_Mgryph_* | This study |
| ∆*mamK_Mgryph_*::*egfp-mamK*_DCbin4_ | Strain with chromosomally encoded EGFP-MamK_DCbin4_ fusion expressed in ∆*mamK_Mgryph_* | This study |
| ∆*mamK_Mgryph_*::*egfp-mamK*_RS-1_ | Strain with chromosomally encoded EGFP-MamK_RS-1_fusion expressed in ∆*mamK_Mgryph_* | This study |
| ∆*mamK_Mgryph_*::*egfp-mamK*_BW-1_ | Strain with chromosomally encoded EGFP-MamK_BW-1_fusion expressed in ∆*mamK_Mgryph_* | This study |
| ∆*mamK_Mgryph_*::*egfp-mamK*_TM-1_ | Strain with chromosomally encoded EGFP-MamK_TM-1_fusion expressed in ∆*mamK_Mgryph_* | This study |
| ∆*mamK_Mgryph_*::*egfp-mamK*_HCH-1_ | Strain with chromosomally encoded EGFP-MamK_HCH-1_fusion expressed in ∆*mamK_Mgryph_* | This study |
| ∆*mamK_Mgryph_*::*egfp-mamK*_MYR-1_ | Strain with chromosomally encoded EGFP-MamK_MYR-1_ fusion expressed in ∆*mamK_Mgryph_* | This study |
| ∆*mamK_Mgryph_*::*egfp-mamK*_LUCA_ | Strain with chromosomally encoded EGFP-MamK_LUCA_ fusion expressed in ∆*mamK_Mgryph_* | This study |
| ∆*mamK_Mgryph_*::*egfp-mad28*_BW-1_ | Strain with chromosomally encoded EGFP-Mad28_BW-1_ fusion expressed in ∆*mamK_Mgryph_* | This study |
| ∆*mamK_Mgryph_*::*egfp-mad28*_HCH-1_ | Strain with chromosomally encoded EGFP-Mad28_HCH-1_ fusion expressed in ∆*mamK_Mgryph_* | This study |
| ∆*mamK_Mgryph_*::*egfp-mad28*_TM-1_ | Strain with chromosomally encoded EGFP-Mad28_TM-1_ fusion expressed in ∆*mamK_Mgryph_* | This study |
| ∆*mamK_Mgryph_*::*mamK-mg-1g50*_MV-1_ | Strain with chromosomally inserted *mamK-mg-1g50*_MV-1_in ∆*mamK_Mgryph_* | This study |
| ∆*mamKY_Mgryph_*::*mamK-mg-1g50*_MV-1_ | Strain with chromosomally inserted *mamK-mg-1g50*_MV-1_in ∆*mamKY_Mgryph_* | This study |
| *E.coli*-P*_tet_*-*egfp*-*mamK_Mgryph_* | *E.coli* with an anhydrotetracycline inducible *egfp*-*mamK_Mgryph_* expression cassette. | This study |
| *E.coli*-P*_tet_*-*egfp*-*mamK*_AMB-1_ | *E.coli* with an anhydrotetracycline inducible *egfp*-*mamK*_AMB-1_ expression cassette. | This study |
| *E.coli*-P*_tet_*-*egfp*-*mamK*_MV-1_ | *E.coli* with an anhydrotetracycline inducible *egfp*-*mamK*_MV-1_ expression cassette. | This study |
| *E.coli*-P*_tet_*-*egfp*-*mamK*_MC-1_ | *E.coli* with an anhydrotetracycline inducible *egfp*-*mamK*_MC-1_ expression cassette. | This study |
| *E.coli*-P*_tet_*-*egfp*-*mamK*_Dcbin4_ | *E.coli* with an anhydrotetracycline inducible *egfp*-*mamK*_Dcbin4_ expression cassette. | This study |
| *E.coli*-P*_tet_*-*egfp*-*mamK*_RS-1_ | *E.coli* with an anhydrotetracycline inducible *egfp*-*mamK*_RS-1_ expression cassette. | This study |
| *E.coli*-P*_tet_*-*egfp*-*mamK*_BW-1_ | *E.coli* with an anhydrotetracycline inducible *egfp*-*mamK*_BW-1_ expression cassette. | This study |
| *E.coli*-P*_tet_*-*egfp*-*mamK*_TM-1_ | *E.coli* with an anhydrotetracycline inducible *egfp*-*mamK*_TM-1_ expression cassette. | This study |
| *E.coli*-P*_tet_*-*egfp*-*mamK*_HCHbin-1_ | *E.coli* with an anhydrotetracycline inducible *egfp*-*mamK*_HCHbin-1_ expression cassette. | This study |
| *E.coli* -P*_tet_*-*egfp*-*mamK*_MYR-1_ | *E.coli* with an anhydrotetracycline inducible *egfp*-*mamK*_MYR-1_ expression cassette. | This study |
| *E.coli*-P*_tet_*-*egfp*-*mamK*_LUCA_ | *E.coli* with an anhydrotetracycline inducible *egfp*-*mamK*_LUCA_ expression cassette. | This study |
| *E.coli*-P*_tet_*-*egfp*-*mad28*_BW-1_ | *E.coli* with an anhydrotetracycline inducible *egfp*-*mad28*_BW-1_ expression cassette. | This study |
| *E.coli*-P*_tet_*-*egfp*-*mad28*_HCHbin1_ | *E.coli* with an anhydrotetracycline inducible *egfp*-*mad28*_HCHbin1_ expression cassette. | This study |
| *E.coli*-P*_tet_*-*egfp*-*mad28*_TM-1_ | *E.coli* with an anhydrotetracycline inducible *egfp*-*mad28*_TM-1_ expression cassette. | This study |

**Table S1D**: Oligonucleotides used in this study. The sites recognized by restriction enzymes are underlined.

| **Primer name** | **sequence 5'-3'** | **Purpose** | **Restriction Enzyme** |
| --- | --- | --- | --- |
|  | **Construction of pBAM-Tn5-P_mamDC45_ based plasmids** | |  |
| **RPA2141** | GGGAATTCCATATGAGTGAAGGTGAAGGCCAG | amplification of *mamK_Mgryph_* | Ndel |
| **RPA2142** | CAGGGATCCTCACTGACCGGAAACGTCACCAAG |  | BamHI |
| **RPA2143** | GGGAATTCCATATGAGTGAAGGTGAAGGCCAGGC | amplification of *mamK*_AMB-1_ | Ndel |
| **RPA2144** | CGCGGATCCTCACGAGCCGGAGACGTCTCCAAG |  | BamHI |
| **RPA2183** | GGAATTCCATATGACCAACCGCGGCCACAAG | amplification of *mamK*_MV-1_ | Ndel |
| **RPA1418** | ATCTTAATAGTCGGTTACGTTGCCGAG |  | - |
| **RPA2179** | GGAATTCCATATGCAGTCTCCTGCAGGTAATG | amplification of *mamK*_MC-1_ | Ndel |
| **RPA1422** | ATCTTAGCCCCCAACGATGTCG |  | - |
| **RPA2147** | GGGAATTCCATATGTCCGGAAGCAACGTGCTGAAC | amplification of *mamK*_RS-1_ | Ndel |
| **RPA2148** | CGCGGATCCTTAATCCTTTCGCAGCTCGTCC |  | BamHI |
| **RPA2153** | CCTGCGAAGCTTAGGAGATCAGCATATGGGAACCACAAAAGCAATCAAC | amplification of *mamK*_BW-1_ | - |
| **RPA2154** | GAACGGTAGGGACCCGGATCCTTATTCGGGGTCGGCAATTTCGTC |  | - |
| **RPA655** | GGGAGTTCCATATGGCTGATGAGAAAGAGCTGAAAG | amplification of *mad28*_BW-1_ | Ndel |
| **RPA654** | CGCGGATCCTTATTCCGCTTCTGTCATTGC |  | BamHI |
|  | **Construction of pBAM-Tn5-P_mamDC45-_egfp-N-terminal based plasmids** | |  |
| **RPA1411** | ATGAGTGAAGGTGAAGGCCAGG | amplification of *mamK_Mgryph_* | - |
| **RPA1412** | ATCTCACTGACCGGAAACGTCAC |  | - |
| **RPA1413** | ATGAGTGAAGGTGAAGGCCAGGC | amplification of *mamK*_AMB-1_ | - |
| **RPA1414** | ATCTCACGAGCCGGAGACGTCTC |  | - |
| **RPA1417** | ATGACCAACCGCGGCCACAAG | amplification of *mamK*_MV-1_ | - |
| **RPA1418** | ATCTTAATAGTCGGTTACGTTGCCGAG |  | - |
| **RPA1421** | ATGCAGTCTCCTGCAGGTAATG | amplification of *mamK*_MC-1_ | - |
| **RPA1422** | ATCTTAGCCCCCAACGATGTCG |  | - |
| **RPA1429** | ATGATTCAGGAAATGGATAAGAC | amplification of *mamK*_DCbin-4_ | - |
| **RPA1430** | ATCTCATTCCCCCATCACATCG |  | - |
| **RPA1423** | ATGTCCGGAAGCAACGTGCTG | amplification of *mamK*_RS-1_ | - |
| **RPA1424** | ATCTTAATCCTTTCGCAGCTCG |  | - |
| **RPA1425** | ATGGGAACCACAAAAGCAATCAAC | amplification of *mamK*_BW-1_ | - |
| **RPA1426** | ATCTTATTCGGGGTCGGCAATTTC |  | - |
| **RPA1435** | ATGGCAAAAACGAAGGTACTTAATATTG | amplification of *mamK*_TM-1_ | - |
| **RPA1436** | ATCTTATCTCTTGCTTACCTCGTC |  | - |
| **RPA1433** | ATGGTTGGTAAGAGGGTTATTAATGTTGG | amplification of *mamK*_HCHbin-1_ | - |
| **RPA1434** | ATCTTAGTTTTTCACCTTACTCTGCCC |  | - |
| **RPA1431** | ATGACAAAAACAAAGATACTTAACATTGG | amplification of *mamK*_MYR-1_ | - |
| **RPA1432** | ATCTTATCGCTTGCTTACTTCGTC |  | - |
| **RPA1441** | ATGACCAAGAAGAAGCTGTATCTGGGCATC | amplification of *mamK*_LUCA_ | - |
| **RPA1442** | ATCTCAGCCCGAGGTGGTGTCGCCC |  | - |
| **RPA372** | ATGGCTGATGAGAAAGAGCTG | amplification of *mad28*_BW-1_ | - |
| **RPA373** | ATCTTATTCCGCTTCTGTCATTGCC |  | - |
| **RPA1437** | ATGGCAGAAGCAAGCGATTATC | amplification of *mad28*_HCHbin-1_ | - |
| **RPA1438** | ATCTTAACGAGACTCTGCCATGG |  | - |
| **RPA1439** | ATGGCGGATGAGTTCAACAAATTTC | amplification of *mad28*_TM-1_ | - |
| **RPA1440** | ATCTTATGCCTCCACGGTCATGG |  | - |
|  | **Sequencing primers for pBAM-Tn5-based plasmids** | |  |
| **RPA684** | CACTTAACGGCTGACATGGG | sequencing primer | - |
| **RPA305** | CAAACTGCAATTTCATCTGATGCTG | seq/screening primer | - |
| **RPA569** | CTTCTTTGGCGGCGGCTTC | sequencing primer | - |
| **RPA1700** | GAATTCCTCGAGCTTTTTCGC | Seq/screening primer |  |
|  | **Construction of pET28a-Mad28_BW-1_ plasmid** | |  |
| **RPA655** | GGGAGTTCCATATGGCTGATGAGAAAGAGCTGAAAG | amplification of *mad28*_BW-1_ | Ndel |
| **RPA654** | CGCGGATCCTTATTCCGCTTCTGTCATTGC |  | BamHI |
| **RPA574** | CATCATCATCACAGCAGCGG | sequencing primer | - |
| **RPA575** | TTAGCAGCCGGATCTCAGTG | sequencing primer | - |
|  | **Construction of pBAM-Tn5-P_mamDC_-*mamK*_MV-1_-oRBS-*mv-1g50*_MV-1_ plasmid** | |  |
| **RPA2274** | CTGCGAAGCTTAGGAGATCAGCATATGACGCAAAGACGGGTAAATAAG | amplification of *mamK*_MV-1_ | - |
| **RPA2275** | CATATGCTGATCTCCTGGTACCTTAATAGTCGGTTACGTTGCC |  | - |
| **RPA2276** | TAAGGTACCAGGAGATCAGCATATGGCGGACAAGAGCACCAACG | amplification of *mv-1g50*_MV-1_ | - |
| **RPA2277** | GAACGGTAGGGACCCGGATCCCTAAACTTTCCACGCCAGCGAAAGC |  | - |
| **RPA2279** | CGATGAACAACTCGAAGCGC | sequencing primer | - |
|  | **Construction of pET24-P_lac_-mCherry-*mad28*_BW-1_-*egfp*-*mamK_Mgryph_* plasmid** | |  |
| **RPA673** | GGAATTGTGAGCGGATAACAATTTCACACAGGAAACACAATGGTGAGCAAGGGCGAGG | amplification of *mCherry-mad28BW-1* from pBBR*-p_tet_-mCherry-mad2*8_BW-1_ | - |
| **RPA674** | CACTCCGTTCGCGTGCTAACAGATCAATCCACAGGTCTATTTATTCCGCTTCTGTCATTGCC |  | - |
| **RPA677** | GCAGCTGGCACGACAGGTTTCCCG | sequencing primer | - |
| **RPA678** | GGAAAGCGGGCAGTGAGC | sequencing primer | - |
| **RPA679** | GAACTTCAGGGTCAGCTTGC | sequencing primer | - |
| **RPA680** | TCCAGCTCGACCAGGATG | sequencing primer | - |
| **RPA681** | GGCATGGACGAGCTGTAC | sequencing primer | - |
| **RPA522** | GAGTTCATGCGCTTCAAGGTG | sequencing primer | - |
| **RPA523** | AGCCGTACATGAACTGAGGG | sequencing primer | - |
| **RPA569** | CTTCTTTGGCGGCGGCTTC | sequencing primer | - |
|  | **Construction of pUT18/pUT18C/pKT25/pKNT25 plasmids** | |  |
| **RPA929** | CTAGTCTAGAGATGGCTGATGAGAAAGAGCTG | amplification of *mad28*_BW-1_ | XbaI |
| **RPA930** | TCCCCCGGGGTTCCGCTTCTGTCATTGC |  | SmaI |
| **RPA931** | CACTTTATGCTTCCGGCTCG | sequencing primer | - |
| **RPA932** | AACTGTTGGGAAGGGCGATC | sequencing primer | - |
| **RPA933** | TTCCACTGACGGCGGATATC | sequencing primer | - |
| **RPA934** | CGGGGCTGGCTTAACTATGC | sequencing primer | - |
| **RPA935** | TTCCACAACAAGTCGATGCG | sequencing primer | - |
| **RPA936** | AAGTTCTCGCCGGATGTACTG | sequencing primer | - |

**Table S1E:** Plasmids used in this study.

| Plasmid | Relevant characteristic (s) | References and/or source | |
| --- | --- | --- | --- |
| pBAM-P*_mamDC_*_45_-*egfp* (N-ter)-HL | Tn5-based integrative plasmid harboring a constitutive promoter P*_mamDC_*_45_-*egfp* (for *N*-terminus fusion) | | (23) |
| pBAM-P*_mamDC_*_45_-*mamK_Mgryph_* | Tn5-based integrative plasmid harboring a constitutive P*_mamDC_*_45_-*mamK_Mgryph_* expression cassette. | | This study |
| pBAM-P*_mamDC_*_45_-*mamK*_AMB-1_ | Tn5-based integrative plasmid harboring a constitutive P*_mamDC_*_45_-*mamK*_AMB-1_ expression cassette. | | This study |
| pBAM-Tn5-P*_mamDC_*_45_-*mamK*_MV-1_ | Tn5-based integrative plasmid harboring a constitutive P*_mamDC_*_45_-*mamK*_MV-1_ expression cassette. | | This study |
| pBAM-P*_mamDC_*_45_-*mamK*_MC-1_ | Tn5-based integrative plasmid harboring a constitutive P*_mamDC_*_45_-*mamK*_MC-1_ expression cassette. | | This study |
| pBAM-P*_mamDC_*_45_-*mamK*_Dcbin4_ | Tn5-based integrative plasmid harboring a constitutive P*_mamDC_*_45_-*mamK*_Dcbin4_ expression cassette. | | This study |
| pBAM-P*_mamDC_*_45_-*mamK*_RS-1_ | Tn5-based integrative plasmid harboring a constitutive P*_mamDC_*_45_-*mamK*_RS-1_ expression cassette. | | This study |
| pBAM-P*_mamDC_*_45_-*mamK*_BW-1_ | Tn5-based integrative plasmid harboring a constitutive P*_mamDC_*_45_-*mamK*_BW-1_ expression cassette. | | This study |
| pBAM-P*_mamDC_*_45_-*mamK*_TM-1_ | Tn5-based integrative plasmid harboring a constitutive P*_mamDC_*_45_-*mamK*_TM-1_ expression cassette. | | This study |
| pBAM-P*_mamDC_*_45_-*mamK*_HCHbin-1_ | Tn5-based integrative plasmid harboring a constitutive P*_mamDC_*_45_-*mamK*_HCHbin-1_ expression cassette. | | This study |
| pBAM-P*_mamDC_*_45_-*mamK*_MYR-1_ | Tn5-based integrative plasmid harboring a constitutive P*_mamDC_*_45_-*mamK*_MYR-1_ expression cassette. | | This study |
| pBAM-P*_mamDC_*_45_-*mamK*_LUCA_ | Tn5-based integrative plasmid harboring a constitutive P*_mamDC_*_45_-*mamK*_LUCA_ expression cassette. | | This study |
| pBAM-P*_mamDC_*_45_-*mad28*_BW-1_ | Tn5-based integrative plasmid harboring a constitutive P*_mamDC_*_45_-*mad28*_BW-1_ expression cassette. | | This study |
| pBAM-P*_mamDC_*_45_-*mad28*_HCHbin1_ | Tn5-based integrative plasmid harboring a constitutive P*_mamDC_*_45_-*mad28*_HCHbin1_ expression cassette. | | This study |
| pBAM-P*_mamDC_*_45_-*mad28*_TM-1_ | Tn5-based integrative plasmid harboring a constitutive P*_mamDC_*_45_-*mad28*_TM-1_ expression cassette. | | This study |
| pBAM-P*_mamDC_*_45_-*egfp*-*mamK_Mgryph_* | Tn5-based integrative plasmid harboring a constitutive P*_mamDC_*_45_-*egfp*-*mamK_Mgryph_* expression cassette. | | This study |
| pBAM-P*_mamDC_*_45_-*egfp*-*mamK*_AMB-1_ | Tn5-based integrative plasmid harboring a constitutive P*_mamDC_*_45_-*egfp*-*mamK*_AMB-1_expression cassette. | | This study |
| pBAM-P*_mamDC_*_45_-*egfp*-*mamK*_MV-1_ | Tn5-based integrative plasmid harboring a constitutive P*_mamDC_*_45_-*egfp*-*mamK*_MV-1_ expression cassette. | | This study |
| pBAM-P*_mamDC_*_45_-*egfp*-*mamK*_MC-1_ | Tn5-based integrative plasmid harboring a constitutive P*_mamDC_*_45_-*egfp*-*mamK*_MC-1_expression cassette. | | This study |
| pBAM-P*_mamDC_*_45_-*egfp*-*mamK*_Dcbin4_ | Tn5-based integrative plasmid harboring a constitutive P*_mamDC_*_45_-*egfp*-*mamK*_DCbin4_ expression cassette. | | This study |
| pBAM-P*_mamDC_*_45_-*egfp*-*mamK*_RS-1_ | Tn5-based integrative plasmid harboring a constitutive P*_mamDC_*_45_-*egfp*-*mamK*_RS-1_ expression cassette. | | This study |
| pBAM-P*_mamDC_*_45_-*egfp*-*mamK*_BW-1_ | Tn5-based integrative plasmid harboring a constitutive P*_mamDC_*_45_-*egfp*-*mamK*_BW-1_ expression cassette. | | This study |
| pBAM-P*_mamDC_*_45_-*egfp*-*mamK*_TM-1_ | Tn5-based integrative plasmid harboring a constitutive P*_mamDC_*_45_-*egfp*-*mamK*_TM-1_ expression cassette. | | This study |
| pBAM-P*_mamDC_*_45_-*egfp*-*mamK*_HCHbin-1_ | Tn5-based integrative plasmid harboring a constitutive P*_mamDC_*_45_-*egfp*-*mamK*_HCHbin-1_ expression cassette. | | This study |
| pBAM-P*_mamDC_*_45_-*egfp*-*mamK*_MYR-1_ | Tn5-based integrative plasmid harboring a constitutive P*_mamDC_*_45_- *egfp*-*mamK*_MYR-1_ expression cassette. | | This study |
| pBAM-P*_mamDC_*_45_-*egfp*-*mamK*_LUCA_ | Tn5-based integrative plasmid harboring a constitutive P*_mamDC_*_45_-*egfp*-*mamK*_LUCA_ expression cassette. | | This study |
| pBAM-P*_mamDC_*_45_-*egfp*-*mad28*_BW-1_ | Tn5-based integrative plasmid harboring a constitutive P*_mamDC_*_45_-*egfp*-*mad28*_BW-1_ expression cassette. | | This study |
| pBAM-P*_mamDC_*_45_-*egfp*-*mad28*_HCHbin1_ | Tn5-based integrative plasmid harboring a constitutive P*_mamDC_*_45_-*egfp*-*mad28*_HCHbin1_ expression cassette. | | This study |
| pBAM-P*_mamDC_*_45_-*egfp*-*mad28*_TM-1_ | Tn5-based integrative plasmid harboring a constitutive P*_mamDC_*_45_-*egfp*-*mad28*_TM-1_ expression cassette. | | This study |
| pBAM-P*_tet_*-*egfp* | Tn5-based integrative plasmid harboring an anhydrotetracycline inducible *egfp* expression cassette. | | (24) |
| pBAM-P*_tet_*-*egfp*-*mamK_Mgryph_* | Tn5-based integrative plasmid harboring an anhydrotetracycline inducible *egfp*-*mamK_Mgryph_* expression cassette. Both proteins are 4-helix linker. | | This study |
| pBAM-P*_tet_*-*egfp*-*mamK*_AMB-1_ | Tn5-based integrative plasmid harboring an anhydrotetracycline inducible *egfp*-*mamK*_AMB-1_ expression cassette. Both proteins are 4-helix linker. | | This study |
| pBAM-P*_tet_*-*egfp*-*mamK*_MV-1_ | Tn5-based integrative plasmid harboring an anhydrotetracycline inducible *egfp*-*mamK*_MV-1_ expression cassette. Both proteins are 4-helix linker. | | This study |
| pBAM-P*_tet_*-*egfp*-*mamK*_MC-1_ | Tn5-based integrative plasmid harboring an anhydrotetracycline inducible *egfp*-*mamK*_MC-1_ expression cassette. Both proteins are 4-helix linker. | | This study |
| pBAM-P*_tet_*-*egfp*-*mamK*_Dcbin4_ | Tn5-based integrative plasmid harboring an anhydrotetracycline inducible *egfp*-*mamK*_Dcbin4_ expression cassette. Both proteins are 4-helix linker. | | This study |
| pBAM-P*_tet_*-*egfp*-*mamK*_RS-1_ | Tn5-based integrative plasmid harboring an anhydrotetracycline inducible *egfp*-*mamK*_RS-1_ expression cassette. Both proteins are 4-helix linker. | | This study |
| pBAM-P*_tet_*-*egfp*-*mamK*_BW-1_ | Tn5-based integrative plasmid harboring an anhydrotetracycline inducible *egfp*-*mamK*_BW-1_ expression cassette. Both proteins are 4-helix linker. | | This study |
| pBAM-P*_tet_*-*egfp*-*mamK*_TM-1_ | Tn5-based integrative plasmid harboring an anhydrotetracycline inducible *egfp*-*mamK*_TM-1_ expression cassette. Both proteins are 4-helix linker. | | This study |
| pBAM-P*_tet_*-*egfp*-*mamK*_HCHbin-1_ | Tn5-based integrative plasmid harboring an anhydrotetracycline inducible *egfp*-*mamK*_HCHbin-1_ expression cassette. Both proteins are 4-helix linker. | | This study |
| pBAM-P*_tet_*-*egfp*-*mamK*_MYR-1_ | Tn5-based integrative plasmid harboring an anhydrotetracycline inducible *egfp*-*mamK*_MYR-1_ expression cassette. Both proteins are 4-helix linker. | | This study |
| pBAM-P*_tet_*-*egfp*-*mamK*_LUCA_ | Tn5-based integrative plasmid harboring an anhydrotetracycline inducible *egfp*-*mamK*_LUCA_ expression cassette. Both proteins are 4-helix linker. | | This study |
| pBAM-P*_tet_*-*egfp*-*mad28*_BW-1_ | Tn5-based integrative plasmid harboring an anhydrotetracycline inducible *egfp*-*mad28*_BW-1_ expression cassette. Both proteins are 4-helix linker. | | This study |
| pBAM-P*_tet_*-*egfp*-*mad28*_HCHbin1_ | Tn5-based integrative plasmid harboring an anhydrotetracycline inducible *egfp*-*mad28*_HCHbin1_ expression cassette. Both proteins are 4-helix linker. | | This study |
| pBAM-P*_tet_*-*egfp*-*mad28*_TM-1_ | Tn5-based integrative plasmid harboring an anhydrotetracycline inducible *egfp*-*mad28*_TM-1_ expression cassette. Both proteins are 4-helix linker. | | This study |
| pBAM-P*_tet_*-*mamK-mg-1g50*_MV-1_ | Tn5-based integrative plasmid harboring a constitutive P*_mamDC_*_45_-*mamK-mg-1g50*_MV-1_ expression cassette. | | This study |
| pET28a | KnR, T7lac, His-Tag N-terminal, β-Gal | | Novagen® |
| pET28a-Mad28_BW-1_ | KnR, T7lac, His-Tag N-terminal- Mad28_BW-1_, β-Gal | | This study |
| pBBR-P*_tet_*-mCherry-*mad28*_BW-1_ | Ptet, KnR, helixlinker with mCherry-*mad28*_BW-1_ | | Awal, unpublished |
| pET24a-plac-*mcherry*-*mad28*_BW-1_-*egfp*-*MamK_Mgryph_* | pET24a based plasmid for co-expression of *mad28*_BW-1_ and *MamK_Mgryph_* | | This study |
| pUT18C | BACTH vector designed to express a given polypeptide fused in frame at its N-terminal end with T18 fragment; ColE1 ori; Amp^R^ | | (25) |
| pUT18 | BACTH vector designed to express a given polypeptide fused in frame at its C-terminal end with T18 fragment; ColE1 ori; Amp^R^ | | (25) |
| pKT25 | BACTH vector designed to express a given polypeptide fused in frame at its N-terminal end with T25 fragment; p15 ori; Km^R^ | | (25) |
| pKNT25 | BACTH vector designed to express a given polypeptide fused in frame at its C-terminal end with T25 fragment; p15 ori; Km^R^ | | (25) |
| pUT18C-zip; pKT25-zip | Derivatives of pUT18C and pKT25 with a 114 bp DNA fragment encoding for a leucine zipper (positive control for two hybrid assays) | | (25) |
| pUT18C-*mamK_Mgryph_* | Two-Hybrid plasmid coding for T18-MamK | | (24) |
| pUT18-*mamK_Mgryph_* | Two-Hybrid plasmid coding for MamK-T18 | | (24) |
| pKT25-*mamK_Mgryph_* | Two-Hybrid plasmid coding for T25-MamK | | (24) |
| pKNT25-*mamK_Mgryph_* | Two-Hybrid plasmid coding for MamK-T25 | | (24) |
| pUT18C-*mamJ_Mgryph_* | Two-Hybrid plasmid coding for T18-MamJ | | (22) |
| pUT18-*mamJ_Mgryph_* | Two-Hybrid plasmid coding for MamJ-T18 | | (22) |
| pKT25-*mamJ_Mgryph_* | Two-Hybrid plasmid coding for T25-MamJ | | (22) |
| pKNT25-*mamJ_Mgryph_* | Two-Hybrid plasmid coding for MamJ-T25 | | (22) |
| pUT18C-*mamY_Mgryph_* | Two-Hybrid plasmid coding for T18-MamY | | (22) |
| pUT18-*mamY_Mgryph_* | Two-Hybrid plasmid coding for MamY-T18 | | (22) |
| pKT25-*mamY_Mgryph_* | Two-Hybrid plasmid coding for T25-MamY | | (22) |
| pKNT25-*mamY_Mgryph_* | Two-Hybrid plasmid coding for MamY-T25 | | (22) |
| pUT18C-*mad28*_BW-1_ | *mad28*_BW-1_ amplified with primers RPA929 & RPA930 cloned into pUT18C via XbaI and SmaI | | This study |
| pUT18- *mad28*_BW-1_ | *mad28*_BW-1_ amplified with primers RPA929 & RPA930 cloned into pUT18 via XbaI and SmaI | | This study |
| pKT25- *mad28*_BW-1_ | *mad28*_BW-1_ amplified with primers RPA929 & RPA930 cloned into pKT25 via XbaI and SmaI | | This study |
| pKNT25-*mad28*_BW-1_ | *mad28*_BW-1_ amplified with primers RPA929 & RPA930 cloned into pKNT25 via XbaI and SmaI | | This study |
|  |  | |  |

References

1. Monteil CL, Perrière G, Menguy N, Ginet N, Alonso B, Waisbord N, Cruveiller S, Pignol D, Lefèvre CT. 2018. Genomic study of a novel magnetotactic Alphaproteobacteria uncovers the multiple ancestry of magnetotaxis. Environ Microbiol 20:4415–4430.

2. Uebe R, Schüler D, Jogler C, Wiegand S. 2018. Reevaluation of the Complete Genome Sequence of Magnetospirillum gryphiswaldense MSR-1 with Single-Molecule Real-Time Sequencing Data. Genome Announc 6.

3. Ji B, Zhang S-D, Arnoux P, Rouy Z, Alberto F, Philippe N, Murat D, Zhang W-J, Rioux J-B, Ginet N, Sabaty M, Mangenot S, Pradel N, Tian J, Yang J, Zhang L, Zhang W, Pan H, Henrissat B, Coutinho PM, Li Y, Xiao T, Médigue C, Barbe V, Pignol D, Talla E, Wu L-F. 2014. Comparative genomic analysis provides insights into the evolution and niche adaptation of marine Magnetospira sp. QH-2 strain. Environ Microbiol 16:525–544.

4. Trubitsyn D, Abreu F, Ward FB, Taylor T, Hattori M, Kondo S, Trivedi U, Staniland S, Lins U, Bazylinski DA. 2016. Draft Genome Sequence of Magnetovibrio blakemorei Strain MV-1, a Marine Vibrioid Magnetotactic Bacterium. Genome Announc 4.

5. Lin W, Zhang W, Paterson GA, Zhu Q, Zhao X, Knight R, Bazylinski DA, Roberts AP, Pan Y. 2020. Expanding magnetic organelle biogenesis in the domain Bacteria. bioRxiv.

6. Monteil CL, Vallenet D, Schüler D, Lefevre CT. 2022. Magnetosome proteins belong to universal protein families involved in many cell processes. Proc Natl Acad Sci U S A.

7. Ji B, Zhang S-D, Zhang W-J, Rouy Z, Alberto F, Santini C-L, Mangenot S, Gagnot S, Philippe N, Pradel N, Zhang L, Tempel S, Li Y, Médigue C, Henrissat B, Coutinho PM, Barbe V, Talla E, Wu L-F. 2017. The chimeric nature of the genomes of marine magnetotactic coccoid-ovoid bacteria defines a novel group of Proteobacteria. Environ Microbiol 19:1103–1119.

8. Schübbe S, Williams TJ, Xie G, Kiss HE, Brettin TS, Martinez D, Ross CA, Schüler D, Cox BL, Nealson KH, Bazylinski DA. 2009. Complete genome sequence of the chemolithoautotrophic marine magnetotactic coccus strain MC-1. Appl Environ Microbiol 75:4835–4852.

9. Geurink C, Lefevre CT, Monteil CL, Morillo-Lopez V, Abreu F, Bazylinski DA, Trubitsyn D. 2020. Complete Genome Sequence of Strain BW-2, a Magnetotactic Gammaproteobacterium in the Family Ectothiorhodospiraceae, Isolated from a Brackish Spring in Death Valley, California. Microbiol Resour Announc 9.

10. Trubitsyn D, Monteil CL, Geurink C, Morillo-Lopez V, Gonzaga Paula de Almeida L, Ribeiro de Vasconcelos AT, Abreu F, Bazylinski DA, Lefevre CT. 2021. Complete Genome Sequence of Strain SS-5, a Magnetotactic Gammaproteobacterium Isolated from the Salton Sea, a Shallow, Saline, Endorheic Rift Lake Located on the San Andreas Fault in California. Microbiol Resour Announc 10.

11. Lefèvre CT, Viloria N, Schmidt ML, Pósfai M, Frankel RB, Bazylinski DA. 2012. Novel magnetite-producing magnetotactic bacteria belonging to the Gammaproteobacteria. ISME J 6:440–450.

12. Lefèvre CT, Trubitsyn D, Abreu F, Kolinko S, Jogler C, de Almeida LGP, de Vasconcelos ATR, Kube M, Reinhardt R, Lins U, Pignol D, Schüler D, Bazylinski DA, Ginet N. 2013. Comparative genomic analysis of magnetotactic bacteria from the Deltaproteobacteria provides new insights into magnetite and greigite magnetosome genes required for magnetotaxis. Environ Microbiol 15:2712–2735.

13. Nakazawa H, Arakaki A, Narita-Yamada S, Yashiro I, Jinno K, Aoki N, Tsuruyama A, Okamura Y, Tanikawa S, Fujita N, Takeyama H, Matsunaga T. 2009. Whole genome sequence of Desulfovibrio magneticus strain RS-1 revealed common gene clusters in magnetotactic bacteria. Genome Res 19:1801–1808.

14. Kolinko S, Richter M, Glöckner F-O, Brachmann A, Schüler D. 2014. Single-cell genomics reveals potential for magnetite and greigite biomineralization in an uncultivated multicellular magnetotactic prokaryote. Environ Microbiol Rep 6:524–531.

15. Lefèvre CT, Frankel RB, Pósfai M, Prozorov T, Bazylinski DA. 2011. Isolation of obligately alkaliphilic magnetotactic bacteria from extremely alkaline environments. Environ Microbiol 13:2342–2350.

16. Lin W, Deng A, Wang Z, Li Y, Wen T, Wu L-F, Wu M, Pan Y. 2014. Genomic insights into the uncultured genus “Candidatus Magnetobacterium” in the phylum Nitrospirae. ISME J 8:2463–2477.

17. Lin W, Paterson GA, Zhu Q, Wang Y, Kopylova E, Li Y, Knight R, Bazylinski DA, Zhu R, Kirschvink JL, Pan Y. 2017. Origin of microbial biomineralization and magnetotaxis during the Archean. Proc Natl Acad Sci U S A 114:2171–2176.

18. Bergeron JRC, Hutto R, Ozyamak E, Hom N, Hansen J, Draper O, Byrne ME, Keyhani S, Komeili A, Kollman JM. 2017. Structure of the magnetosome-associated actin-like MamK filament at subnanometer resolution. Protein Sci 26:93–102.

19. Wiegand S, Jogler M, Kohn T, Awal RP, Oberbeckmann S, Kesy K, Jeske O, Schumann P, Peeters SH, Kallscheuer N, Strauss M, Heuer A, Jetten MSM, Labrenz M, Rohde M, Boedeker C, Engelhardt H, Schüler D, Jogler C. 2019. The novel shapeshifting bacterial phylum Saltatorellota. bioRxiv.

20. Schultheiss D, Kube M, Schüler D. 2004. Inactivation of the flagellin gene flaA in Magnetospirillum gryphiswaldense results in nonmagnetotactic mutants lacking flagellar filaments. Appl Environ Microbiol 70:3624–3631.

21. Katzmann E, Scheffel A, Gruska M, Plitzko JM, Schüler D. 2010. Loss of the actin-like protein MamK has pleiotropic effects on magnetosome formation and chain assembly in Magnetospirillum gryphiswaldense. Mol Microbiol 77:208–224.

22. Toro-Nahuelpan M, Giacomelli G, Raschdorf O, Borg S, Plitzko JM, Bramkamp M, Schüler D, Müller F-D. 2019. MamY is a membrane-bound protein that aligns magnetosomes and the motility axis of helical magnetotactic bacteria. Nat Microbiol 4:1978–1989.

23. Awal RP, Lefevre CT, Schüler D. Functional expression of foreign magnetosome genes in the alphaproteobacterium Magnetospirillum gryphiswaldense. MBio https://doi.org/10.1128/mbio.03282-22.

24. Pfeiffer D, Toro-Nahuelpan M, Awal RP, Müller F-D, Bramkamp M, Plitzko JM, Schüler D. 2020. A bacterial cytolinker couples positioning of magnetic organelles to cell shape control. Proc Natl Acad Sci U S A 117:32086–32097.

25. Karimova G, Ullmann A, Ladant D. 2001. Protein-protein interaction between Bacillus stearothermophilus tyrosyl-tRNA synthetase subdomains revealed by a bacterial two-hybrid system. J Mol Microbiol Biotechnol 3:73–82.
